# Device-measured physical activity and adiposity in schoolchildren: a 30-week follow-up study

**DOI:** 10.1186/s12916-025-04161-4

**Published:** 2025-06-02

**Authors:** Eva Rodríguez-Gutiérrez, Vicente Martínez-Vizcaíno, Irene Sequí-Domínguez, Sergio Núñez de Arenas-Arroyo, Pontus Henriksson, Ángel Herraiz-Adillo, Ana Torres-Costoso

**Affiliations:** 1 Health and Social Research Center, Universidad de Castilla-La Mancha, Cuenca, Spain; 2 Research Network On Chronicity, Primary Care and Health Promotion (RICAPPS), Cuenca, Spain; 3 Facultad de Ciencias de La Salud, Universidad Autónoma de Chile, Talca, Chile; 4 Department of Health, Medicine and Caring Sciences, Linköping University, Linköping, Sweden; 5 Facultad de Fisioterapia y Enfermería, Universidad de Castilla-La Mancha, Toledo, Spain

**Keywords:** Children, Daily steps, Physical activity, Adiposity, Obesity

## Abstract

**Introduction:**

Physical inactivity is a key risk factor for childhood obesity, but there is a lack of evidence based on long-term assessments examining daily step patterns and their association with adiposity parameters. Therefore, the objectives of this study are to examine the device-measured physical activity patterns during the complete week, weekdays, and weekends over 30 weeks in schoolchildren, and to assess the association of physical activity patterns and adherence to daily steps recommendations with adiposity parameters (body mass index (BMI), body fat percentage (BF%), and waist circumference).

**Methods:**

We conducted a follow-up study involving 338 children (55% girls, mean age 11.0 years) from six public primary schools in Cuenca, Spain. Daily steps were measured using the Xiaomi Mi Band 3 Smartwatch over 30 weeks. BMI, BF%, and waist circumference were assessed in the final week of follow-up. Analysis of covariance models and restricted cubic splines examined the dose–response relationship between daily steps (complete week, weekdays, and weekends) and adiposity parameters. Multivariate mixed-effect linear analyses examined the associations of 1000 steps/day increment and adiposity parameters.

**Results:**

Children averaged 861 more steps/day on weekdays compared to weekends. We observed an inverse association between daily steps and adiposity parameters, particularly in those who achieved more than 12,000 steps/day and met the daily step recommendations > 40% of the days (*p* < 0.05), although this was only found in boys. An increase of 1000 steps/day on weekdays was associated with reductions in BMI, BF%, and waist circumference (unstandardised *β* coefficients were − 0.17, − 0.36, and − 0.59, respectively; *p* < 0.05). Sensitivity analyses confirmed that longer monitoring periods provided stronger associations between physical activity and adiposity parameters.

**Conclusions:**

Higher levels of daily steps, especially on weekdays, were associated with lower adiposity in childhood.

**Supplementary Information:**

The online version contains supplementary material available at 10.1186/s12916-025-04161-4.

## How this study might affect research, practice or policy

This study supports the hypothesis that higher levels of daily steps, especially on weekdays, could protect against increased adiposity in childhood. This suggests that the use of this objective measure of physical activity may be a useful strategy for obesity prevention in Spanish children.

## What’s known on this subject

Physical inactivity is a leading factor in childhood obesity, and a higher number of daily steps is associated with better adiposity parameters. However, previous studies focus on short-term assessments of children’s physical activity.

## What this study adds

Long-term monitoring shows that children taking over 12,000 steps daily, particularly on weekdays, have lower adiposity levels. Boys especially benefit, with reduced body fat and BMI.

## Background

Childhood obesity has become a significant global concern [1], with Spain reporting one of the highest obesity rates in Europe (around 40%) according to the latest World Health Organization European Childhood Obesity Surveillance [2]. This global epidemic has important consequences, including adverse health and psychosocial outcomes in childhood and adolescence [3, 4], and an increased risk of non-communicable diseases and early mortality in adulthood [5].

One of the main factors contributing to the current obesity epidemic is the high prevalence of physical inactivity among children [6, 7]. A useful tool to combat this is to measure the number of daily steps in a simple and motivational way using consumer-wearable activity trackers [8]. These devices provide reliable data [9], facilitating comparisons across studies and helping to identify global PA patterns [10].

Children should accumulate 60 min of moderate-to-vigorous PA daily, equivalent to 13,000–15,000 steps for boys and 11,000–12,000 steps for girls [11, 12]. Meeting these step recommendations has been associated with a lower likelihood of having overweight [13]. However, it is necessary to confirm whether following these step recommendations, and also whether the daily step patterns (since children are more likely to be physically active on school days than on the weekend [14]), have consequences on children’s adiposity outcomes.

Higher number of daily steps is associated with better adiposity parameters, such as lower body mass index (BMI), body fat percentage (BF%), and waist circumference (WC) in children [13, 15–17]. However, most research has relied on short-term data, usually 7 days or even less [18]. The short assessment period may introduce biases in measuring PA behaviour due to the Hawthorne effect [19], where participants change their behaviour because they are aware that they are being monitored [20]. Thus, it is necessary to measure daily steps over a longer period, including weekdays and weekends, to obtain a more accurate and natural representation of PA patterns.

In the same vein, it has been suggested that short-term or seasonal studies may not capture the full complexity of variability in children’s activity levels, as both environmental factors and seasonality influence children’s daily steps throughout a school year [21]. Furthermore, a previous systematic review assessing the association of daily steps and adiposity in children indicated the need for prospective studies and highlighted the challenges posed by heterogeneity, which precludes the possibility of conducting a dose–response analysis [18]. Consequently, prospective studies with longer follow-up duration may be required to assess a potential dose–response relationship between daily steps on weekdays and weekends and adiposity parameters.

Therefore, the objectives of this study are: 1) to examine the device-measured PA patterns over a 30-week period in schoolchildren; and 2) to evaluate the association between daily steps taken during the complete week, weekdays, and weekends, and the adherence to daily step recommendations over 30 weeks, with adiposity parameters (BMI, BF%, and WC) in schoolchildren.

## Methods

### Study design and participants

This follow-up study from the e-MOVI project [22, 23] was conducted in accordance with the STROBE guidelines (Additional file 1: Table S1).

A total of 1049 children aged 9 to 13 years old from six public primary schools in the province of Cuenca, Spain, were invited to participate. To be included in the study, participants had to meet the following inclusion criteria: (1) literacy in Spanish (or Spanish sign language); (2) no serious learning difficulties or physical or mental disorders that would prevent participation in the e-MOVI project, as identified by parents and teachers; (3) no material allergies affecting smart wristband use; and (4) parental or legal guardian consent. A total of 745 schoolchildren were ultimately enrolled in the study. Of these, 405 were excluded because they had less than 18 weeks of daily step data. As a result, all the present analyses were performed with 338 children with complete data (Additional file 1: Fig. S1).

### Study variables

The study variables were measured by trained researchers in accordance with standardised conditions. The daily step data collection started in October–November 2022 and ended in May–June 2023, and each child was followed up for 30 weeks (Additional file 1: Fig. S2). In the final week of daily step monitoring, data on adiposity parameters was collected.

#### Exposures: daily steps

Participants wore a Xiaomi Mi Band 3 on their non-dominant wrist to measure their daily steps. The device measures 46.9 × 17.9 × 12 mm and weighs 20 g. It incorporates a tri-axial accelerometer as its primary mechanism for detecting movement and quantifying the number of steps taken.

This device has presented good validity for measuring daily steps in adolescents [9], as well as its updated version (Xiaomi Mi Band 5) in children [24]. Additionally, before the study started, we tested the accuracy of the Xiaomi Mi Band 3 on a sub-sample of participants using a treadmill protocol. Participants completed up to ten 3-min sets at 0% incline, with speed increasing from 0.8 km/h to 0.8 km/h per set. Participants were encouraged to move naturally, and the test ended when either the participant or the investigator decided to stop. Steps counted by the device were compared with manual counts using video recordings of the feet as a backup, and an intraclass correlation of 0.93 was found, indicating excellent agreement.

Parents or legal guardians were instructed to sync the Xiaomi Mi Band 3 with smartphones via Bluetooth, using the proprietary app provided by Xiaomi. The app automatically transmits and aggregates the daily step data to a central server. According to Xiaomi’s privacy policy, direct access to the data was not possible. Therefore, the children recorded their daily steps in a logbook, which was collected weekly at the school by a member of the research team. The mean daily step count for each participant (complete week) was calculated using data from at least 18 weeks, considering only weeks with a minimum of 4 days of records, including at least one weekend day. The number of steps taken on weekdays (Monday to Friday) was calculated in a similar way, requiring at least four weekdays of data per week, and for weekend steps (Saturday to Sunday) a minimum of 1 day was required. To consider a day as a valid day, wear time had to be greater than 12 h [25](as verified by activity logs). This procedure was chosen to minimise the risk of underestimating step counts due to periods of nonwear.

The percentage of days meeting step recommendations was calculated using the guidelines set out by Tudor-Locke for children aged 6–11 years old (≥ 11,000 for girls and ≥ 13,000 for boys) [12]. It was calculated over the entire follow-up period, based on the total number of valid days. For each child, the percentage was obtained by dividing the number of days on which the recommended step threshold was met by the total number of days with valid step data over a 30-week period and then multiplying by 100.

#### Outcomes

##### Body mass index

Height and weight were measured twice non-consecutively during the same measurement process, with the children lightly dressed and without shoes. The mean of the two measurements was used to calculate BMI as weight in kilograms divided by the square of the height in metres (kg/m^2^). Height was measured to the nearest millimetre using a wall-mounted stadiometer (SECA 222, Vogel and Halke, Hamburg, Germany), with the spine aligned with the stadiometer and the head positioned on the Frankfurt horizontal. Weight was measured to the nearest 100 g using a calibrated digital scale (SECA 861; Vogel & Halke, Hamburg, Germany).

##### Body fat percentage

The BF% was estimated using an eight-electrode Tanita Segmental-418 bioimpedance analysis system (Tanita Corp., Tokyo, Japan), valid for assessing whole body composition in school-aged children [26]. The mean of two measurements taken in the morning under controlled temperature and humidity conditions, with the child barefoot, fasting, and after urinating was used.

##### Waist circumference

The WC (centimetres) was calculated as the mean of two measurements taken with non-elastic tape at the waist (at the midpoint between the last rib and the iliac crest) at the end of a normal expiration.

#### Covariates

To identify the minimum sufficient adjustment set (MSAS) for the total effect of daily steps on adiposity parameters, we constructed a directed acyclic graph (DAG), based on prior scientific literature [10, 19] using the online tool DAGitty [27]. The covariates age, sex, number of weeks with step data, dietary intake, socioeconomic status, and pubertal status were identified as the MSAS (Additional file 1: Fig. S3).

All covariates except the number of weeks with step data were assessed at baseline. To assess pubertal status, the Tanner questionnaire [28] was sent to parents or legal guardians, but it was not included in the analyses due to the high non-response rate. A validated questionnaire was also sent to parents to assess maternal education [29], which is widely recognised as an indicator of socioeconomic status [30]. It is strongly associated with children’s cognitive development and is a key predictor of other family resources that strongly predict child well-being: economic insecurity, family structure, and maternal depression [31]. The responses of the questionnaire were categorised into “no studies or primary studies”, “secondary studies”, “high school”, and “university studies”. On the other hand, the only diet-related measured covariate was adherence to the Mediterranean diet and was therefore used as a potential confounding variable. It was evaluated using the Mediterranean Diet Quality Index for Children and Teenagers (KIDMED) index [32], which is based on a 16-item test ranging from 0 to 12 about the consumption of fast food, sweets, and soft drinks, fruit and vegetables, and fish and pulses weekly, with affirmative or negative answers. The total score is then categorised into three levels: low (≤ 3 points), moderate (4–7 points), and high (≥ 8 points).

Nevertheless, due to missing data, only age, sex, and number of weeks with step data were considered in the models as confounders, and a sensitivity analysis was carried out by adding maternal education and adherence to the Mediterranean diet.

### Statistical analysis

We examined the data for outliers and applied a winsorisation to the 98th percentile for BMI, BF%, and WC to limit their influence on the analyses. We assessed the normality of the continuous variable distributions using Kolmogorov–Smirnov tests and normal probability plots. We used Student’s *t*-test for continuous variables and chi-square tests to describe the study sample’s characteristics by sex and to compare those included versus those excluded from the analyses, and those with at least 1 week of step data versus those with at least 18 weeks of step data. Mean daily steps were categorised into tertiles (rounded to the nearest 1000 steps/day) for complete week: < 9000 steps/day, 9000–12,000 steps/day, and > 12,000 steps/day. We also categorised the percentage of days meeting daily step recommendations into tertiles as follows: < 20%, 20–40%, > 40%.

Student’s *t* test was used to test the mean differences and their 95% CI between daily weekday steps and daily weekend steps, by sex, daily steps (complete week) categories, and schools that participated in the project. A cross-frequency table was constructed, and the weighted Cohen’s kappa was calculated to determine the match of classification into the categories of daily steps on weekdays and weekends.

To examine the dose–response association between daily steps indicators and adiposity parameters, we used analysis of covariance (ANCOVA) with two levels of adjustment: unadjusted (Model 0) and adjusted for age, sex, and number of weeks with step data (Model 1). Additionally, restricted cubic splines were used for examining non-linear relationships, with four knots set at the 5th, 35th, 65th, and 95th percentiles of the daily step distribution, within linear regression models [33]. Four knots were chosen to provide an adequate model fit and to ensure a good balance between flexibility and loss of precision due to overfitting in a small sample, using the positions suggested by Harrel et al. [33]. Moreover, we estimated a mixed-effect linear regression model to assess the linear association between 1000 steps/day increment and adiposity parameters, considering the daily steps indicators as fixed-effect factors and the schools that participated in the project as the random-effects factor. An initial unadjusted model was estimated, followed by a model controlling for age, sex, and number of weeks with step data.

First-order interaction parameters were tested between daily steps and BMI, BF%, and WC, by sex. In addition, due to the limited number of studies in diverse populations and the biological and physiological differences between boys and girls [34], we conducted analyses stratified by sex.

Finally, we performed sensitivity analyses by modifying the covariates included in the ANCOVA models. Firstly, we included maternal education and adherence to the Mediterranean diet as confounding variables in adjustment model 1, in addition to age, sex, and number of weeks with step data. Secondly, ANCOVAs were performed by including subjects with 12 or more weeks of daily step data, as there is substantial evidence supporting the efficacy of exercise interventions of at least 12 weeks’ duration in reducing adiposity in children and adolescents [35, 36]. In addition, sensitivity analyses were performed using LOESS (locally estimated scatterplot smoothing) regression to assess the impact of the number of weeks of follow-up accumulated (1 to 24 weeks) and the number of weeks with step data on the partial correlation coefficients between daily steps and adiposity parameters. In this analysis, both participants included in previous analyses and those excluded due to having less than 18 weeks of daily step data for the whole week, weekdays, and weekends were considered.

Analyses were conducted using the statistical software package IBM SPSS Statistics 29.0 (SPSS, Inc., Chicago, IL, USA) and StataSE v.15 (StataCorp, College Station, TX, USA). The statistical significance was set at two-tailed *p* < 0.05.

## Results

### Descriptive data

The study population comprised 338 schoolchildren (mean age 11.0 years, 95% CI: 10.9, 11.1), of whom 186 (55%) were girls and 105 (31%) were classified as having overweight or obesity. Participants had a mean daily step count of 10,823 steps, with 38.6% of days meeting the recommended daily steps. The mean number of weeks for which the children provided valid step data was 23. Boys exhibited a higher daily step count compared to girls, while girls had a higher BF% (*p* < 0.05) (Table 1).

**Table 1:** Characteristics of participants by sex

	**Total (*n* = 338)**	**Girls (*n* = 186)**	**Boys (*n* = 152)**	***p*-value**
Age (years)	11.0 (10.9, 11.1)	11.0 (10.9, 11.1)	11.0 (10.9, 11.1)	0.992
Height (cm)	147.1 (146.1, 148.1)	147.7 (146.3, 149.1)	146.4 (145.0, 147.8)	0.198
Weight (kg)	41.7 (40.5, 42.8)	42.4 (40.8, 44.0)	40.8 (39.2, 42.4)	0.182
**Daily steps**				
Mean daily steps (complete week)	10,823 (10,500, 11,146)	9848 (9,465, 10,231)	12,017 (11,536, 12,498)	< 0.001
Mean daily weekdays steps	11,075 (10,750, 11,392)	10,057 (9,686, 10,428)	12,320 (11,844, 12,797)	< 0.001
Mean daily weekend steps	10,214 (9,860, 10,569)	9455 (9,024, 9,886)	11,144 (10,592, 11,696)	< 0.001
Mean difference between daily weekdays steps and daily weekend steps	861 (673, 1,050)*	602 (387, 817)*	1176 (856,1,496)*	-
Days meeting daily step recommendations (%)^a^	31.6	29.6	34.2	0.049
**Body composition**				
Body mass index (kg/m^2^)	19.0 (18.6, 19.4)	19.1 (18.6, 19.6)	18.9 (18.4, 19.4)	0.448
Body fat (%)	24.8 (24.1, 25.4)	26.60 (25.8, 27.4)	22.5 (21.5, 23.5)	< 0.001
Waist circumference (cm)	64.8 (63.7, 65.8)	64.5 (63.1, 65.9)	65.1 (63.6, 66.6)	0.588
Overweight/obesity (%)	31.2	32.8	28.9	0.447

Data are presented by mean and 95% confidence interval. * *p* < 0.001

^a^The percentage of days children met the daily step recommendations was calculated considering the daily step recommendations established by Tudor-Locke for children aged 6 to 11 years old, which were ≥ 11,000 steps/day in girls and ≥ 13,000 steps/day in boys [12]

There were no differences between those included in the analyses versus those excluded except for the percentage of girls (55% girls vs. 45% boys) (Additional file 1: Table S2), as well as when comparing those with at least 1 week of step data versus those with at least 18 weeks of step data (Additional file 1: Table S3).

### Differences in daily steps on weekdays and weekends

Children took more daily steps on weekdays than on weekends, regardless of sex, daily step category, and school where the project was conducted (Table 1 and Additional file 1: Table S4). Furthermore, 65% of children (67% boys and 63% girls) remained in the same category of daily steps (Additional file 1: Table S5).

### Association of daily steps indicator with adiposity parameters

No significant interactions were observed between daily steps and BMI, BF%, and WC by sex.

The ANCOVA models (Table 2 and Additional file 1: Fig. S4) revealed that children who accumulated > 12,000 steps/day during the complete week, weekdays, and weekends, and met daily step recommendations on > 20% of days, had lower BF% and WC (*p* < 0.05). In addition, those children who walked > 12,000 steps/day on weekdays also had lower BMI (*p* < 0.05). By sex, girls who took > 12,000 steps/day on weekdays had a smaller WC, and those who met the daily step recommendations on at least > 20% of the days showed a lower BMI and WC (*p* < 0.05) (Additional file 1: Table S6). For boys, those who took > 12,000 steps/day during the week and on weekdays and met the daily step recommendations > 40% of the days had also a lower BF% and WC (Additional file 1: Table S7).

**Table 2:** Analysis of covariance of adiposity parameters by categories of mean daily steps in a complete week, on weekdays, and on weekends, and percentage of days meeting daily step recommendations

		n	**Body mass index**		**% Body fat**		**Waist circumference**	
			M0	M1	M0	M1	M0	M1
**Mean daily steps (complete week)**	Low (L) <9000	105	19.39 (18.73, 20.04)	19.33 (18.66, 20.00)	26.35 (25.17, 27.53)^H^	25.64 (24.45, 26.82)^H^	66.13 (64.32, 67.94)^H^	66.31 (64.5, 68.2)^H^
	Medium (M) 9000–12,000	119	19.15 (18.54, 19.77)	19.25 (18.63, 19.87)	25.38 (24.27, 26.49)^H^	25.21 (24.13, 26.30)	65.34 (63.63, 67.04)	65.78 (64.1, 67.4)^H^
	High (H) >12,000	114	18.49 (17.86, 19.11)	18.44 (17.79, 19.09)	22.66 (21.52, 23.79)^L,M^	23.49 (22.35, 24.64)^L^	62.88 (61.14, 64.61)^L^	62.25 (60.46, 64.03)^L,M^
	*p*-value		0.124	0.125	**<0.001**	**0.031**	**0.028**	**0.004**
**Mean daily weekdays steps**	Low (L) <9000	87	19.62 (18.91, 20.34)^H^	19.59 (18.86, 20.33)^H^	26.45 (25.16, 27.73)^H^	25.81 (24.53, 27.10)^H^	66.66 (64.67, 68.64)^H^	66.87 (64.86, 68.88)^H^
	Medium (M) 9000–12,000	132	19.12 (19.54, 19.70)	19.23 (18.64, 19.82)	25.75 (24.70, 29.80)^H^	25.43 (24.39, 26.47)^H^	65.28 (63.67, 66.90)	65.88 (64.26, 67.49)^H^
	High (H) >12,000	119	18.42 (17.81, 19.03)^L^	18.32 (17.68, 18.96) ^L^	22.44 (21.34, 23.54)^L,M^	23.26 (22.13, 24.39)^L,M^	62.78 (61.08, 64.47)^L^	61.97 (60.21, 63.73)^L,M^
	p-value		**0.037**	**0.034**	**<0.001**	**0.007**	**0.010**	**<0.001**
**Mean daily weekend steps**	Low (L) <9000	132	19.37 (18.79, 19.95)	19.37 (18.78, 19.96)	26.29 (25.23, 27.35) ^H^	25.70 (24.66, 26.75) ^H^	65.84 (64.22, 67.46)	66.12 (64.48, 67.75)^H^
	Medium (M) 9000–12,000	103	18.91 (18.24, 19.58)	18.96 (18.29, 19.63)	24.71 (23.51, 25.92)	24.88 (23.71, 26.04)	64.93 (63.08, 66.79)	65.06 (63.23, 66.90)
	High (H) >12,000	103	18.62 (17.96, 19.28)	18.57 (17.90, 19.24)	22.87 (21.67, 24.06)^L^	23.46 (22.28, 24.63)^L^	63.19 (61.37, 65.02)	62.71 (60.88, 64.55) ^L^
	*p*-value		0.232	0.225	**<0.001**	**0.022**	0.102	**0.027**
**% Days meeting daily step recommendations**	Low (L) <20%	117	19.58 (18.97, 20.19)	19.54 (18.92, 20.16)	26.36 (25.24, 27.48) ^M,H^	26.36 (25.22, 27.50) ^M,H^	66.87 (65.17, 68.57) ^M,H^	66.82 (65.12, 68.52)^M,H^
	Medium (M) 20–40%	105	18.58 (17.94, 19.22)	18.59 (17.95, 19.23)	24.32 (23.14, 25.50)^L^	24.32 (23.14, 25.50) ^L^	63.55 (61.76, 65.34) ^L^	63.55 (61.78, 65.32) ^L^
	High (H) >40%	116	18.80 (18.19, 19.41)	18.83 (18.21, 19.45)	23.58 (22.45, 24.71) ^L^	23.59 (22.45, 24.73) ^L^	63.73 (62.03, 65.43) ^L^	63.77 (62.06, 65.48) ^L^
	*p*-value		0.070	0.097	**0.002**	**0.003**	**0.012**	**0.016**

Data are presented as mean and 95% confidence interval. The values in bold indicate statistical significance at p 0.05. Model 0 (M0): unadjusted data analysis. Model 1 (M1): controlling for age, sex, and number of weeks with step data. Superscript letter indicates statistical significance (p 0.05) between categories for post-hoc tests using the Bonferroni comparisons

**Fig. 1:**
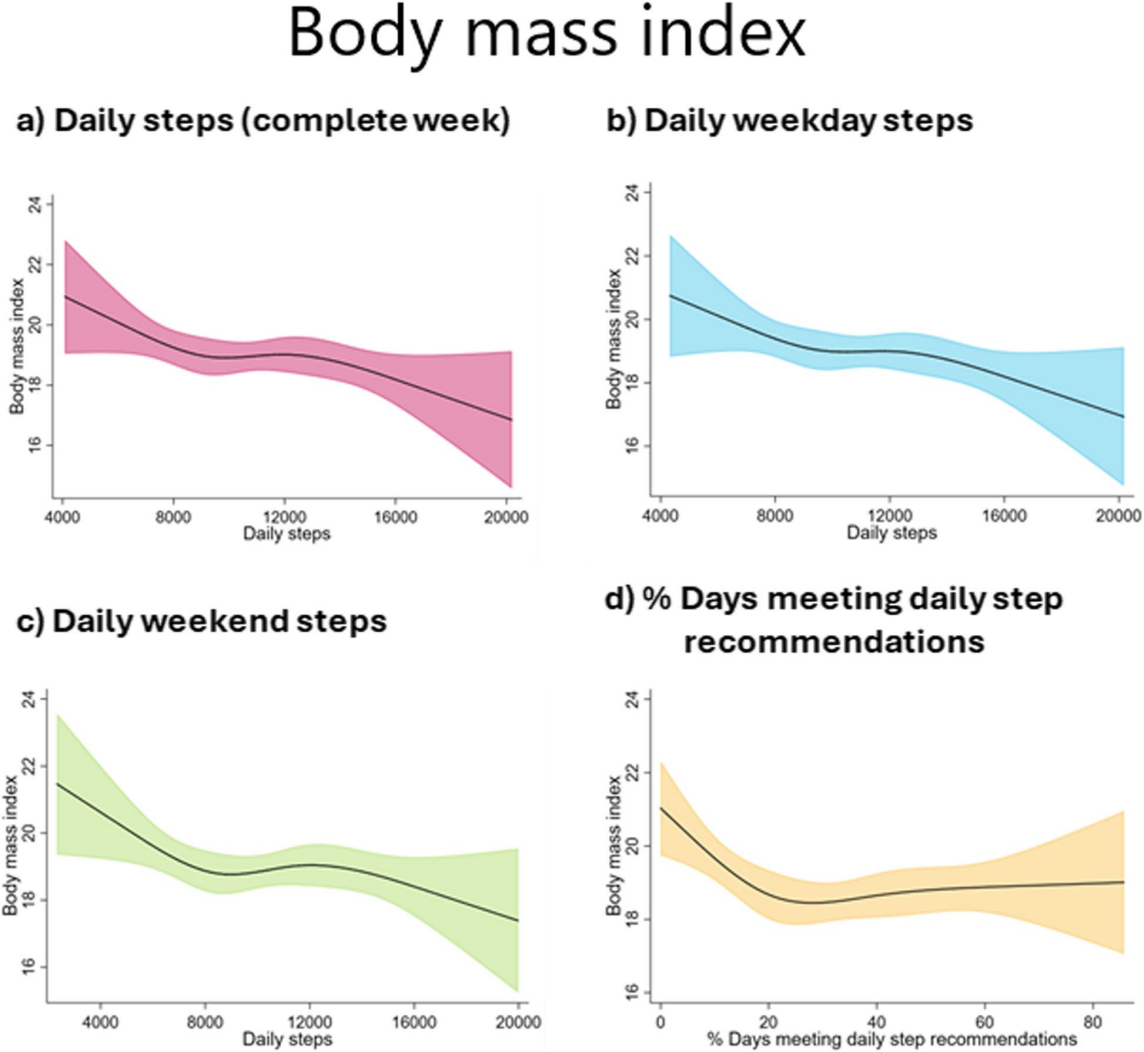
Restricted cubic splines with 95% confidence interval for the association of daily steps and percentage of days meeting daily step recommendations with body mass index (kg/m^2^). The model is adjusted for age and number of weeks with step data

**Fig. 2:**
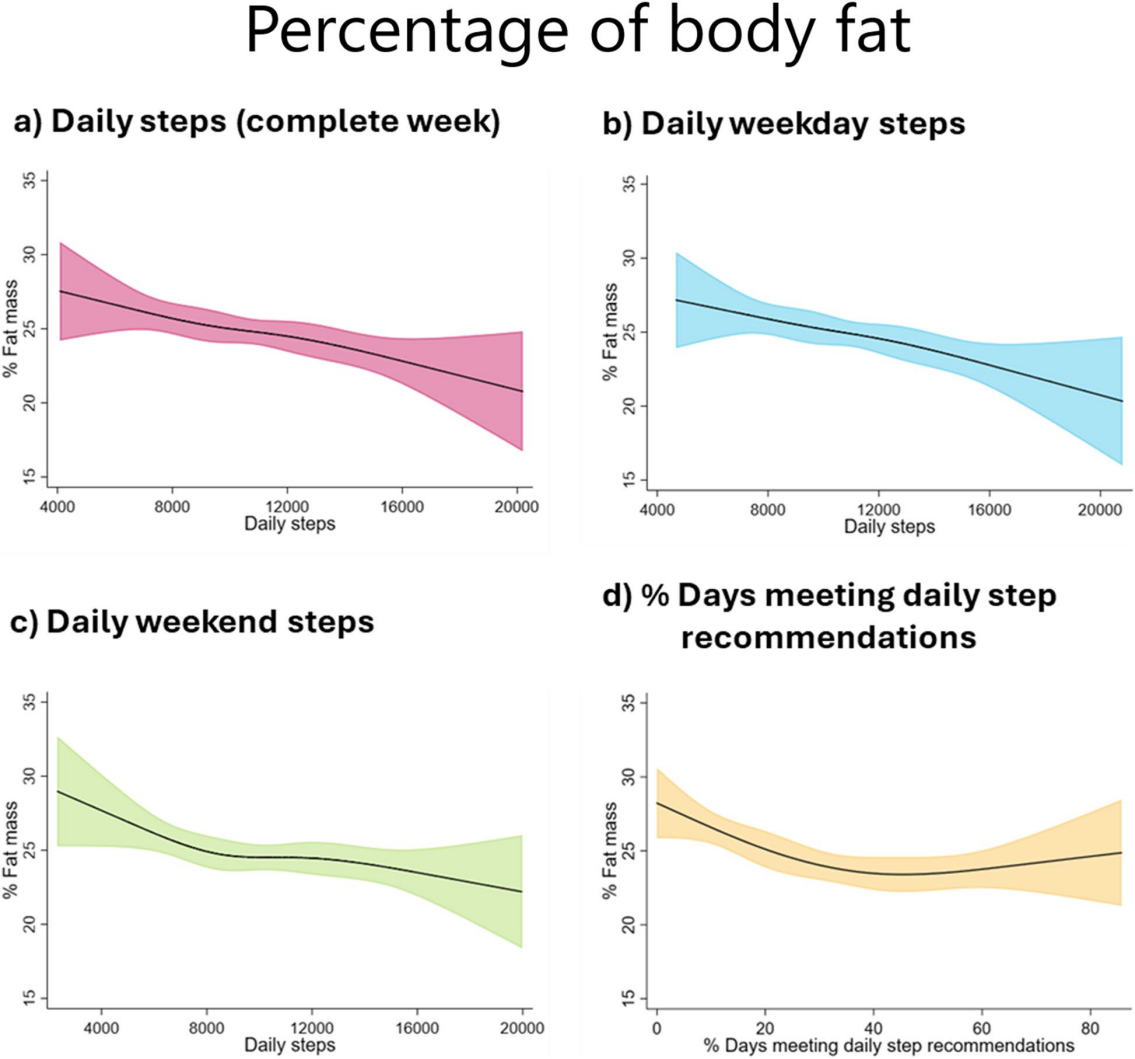
Restricted cubic splines with 95% confidence interval for the association of daily steps and percentage of days meeting daily step recommendations with percentage of body fat. The model is adjusted for age and number of weeks with step data

**Fig. 3:**
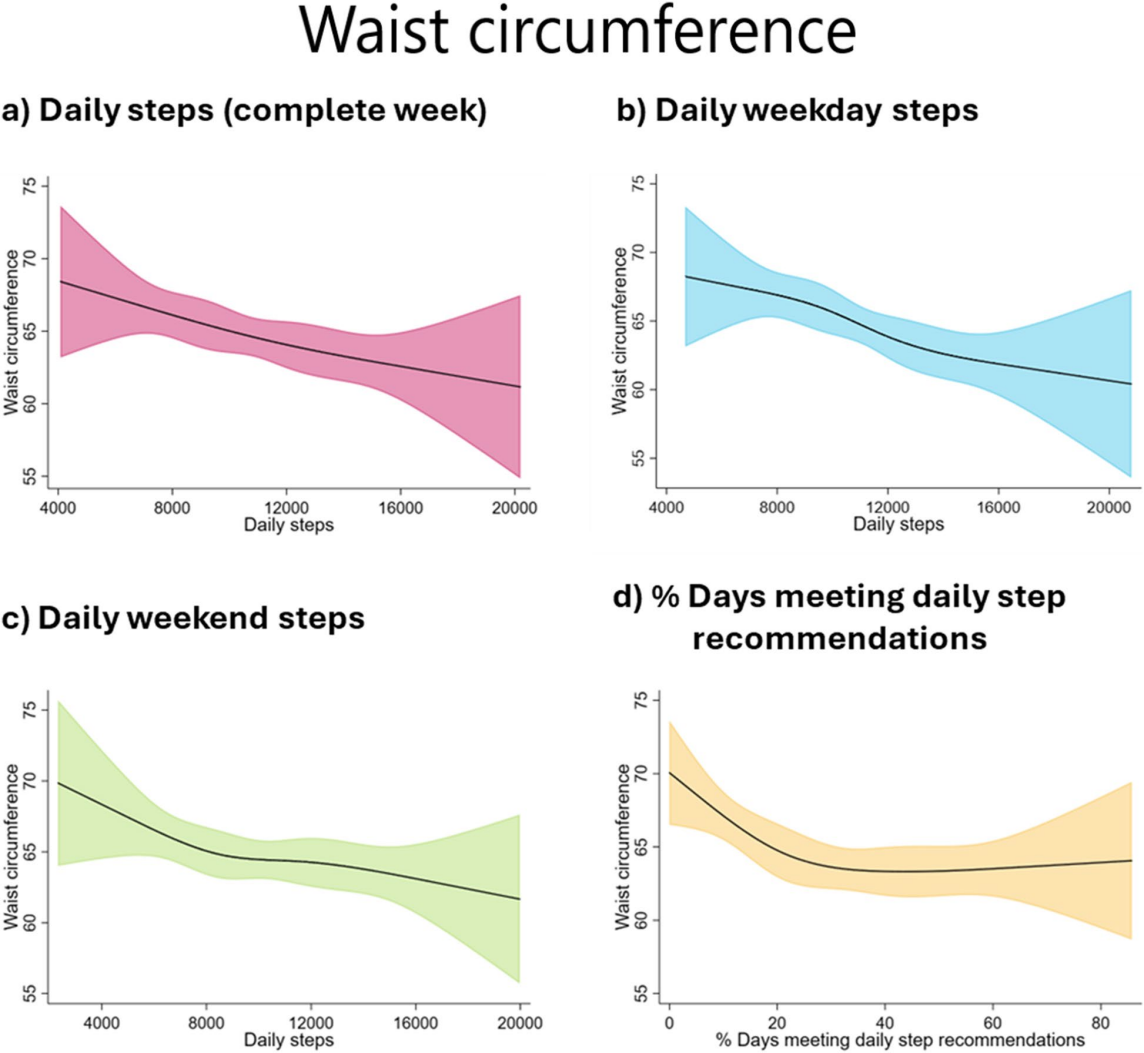
Restricted cubic splines with 95% confidence interval for the association of daily steps and percentage of days meeting daily step recommendations with waist circumference (cm). The model is adjusted for age and number of weeks with step data

Restricted cubic splines indicated a trend towards inverse associations of daily steps with all the adiposity parameters (Figs. 1, 2, and 3). The results of the sex-specific analyses (Additional file 1: Figs. S5–S7) indicated that girls showed an inverse of daily step count and adiposity parameters, stabilising at around 10,000–12,000 steps/day for daily steps taken during the week and at around 30% of days meeting daily recommendations. In boys, a negative association was observed between all daily step indicators and the adiposity parameters.

An increase of 1000 steps/day during the complete week, weekdays, and weekends was associated with the adiposity parameters, except the increase of 1000 steps/day on weekends and BMI and BF%. In boys, but not in girls, the associations remained significant only for the increase of 1000 steps/day during the week and weekdays with all adiposity parameters (Additional file 1: Table S8).

Finally, the results of the sensitivity analyses confirmed that when adherence to the Mediterranean diet and maternal education were added as covariates, the associations disappeared, except for the association of daily steps on weekdays and adherence to daily steps recommendations with WC (Additional file 1: Table S9). It should be noted that the sample size was reduced by almost half (*n* = 175). On the other hand, with a minimum of 12 weeks of daily step data, the association between daily steps and adiposity parameters was consistent. However, there was an exception for adherence, where children who met > 40% of daily step recommendations showed the lowest levels of adiposity (see Additional file 1: Table S10). Regarding the partial correlations between daily steps and adiposity measures, these improved with longer follow-up periods and more weeks of step data collected (Additional file 1: Figs. S8 and S9).

## Discussion

Most evidence linking usual PA and cardiometabolic parameters comes from studies including short-term monitoring of PA, typically 7 days or less. In contrast, our study aimed to examine the device-measured PA patterns over a 30-week period and to assess the association between daily steps and adiposity parameters in schoolchildren. Our findings indicate that children averaged 10,823 steps/day, with boys taking 12,017 steps and girls 9848 steps. Daily steps were significantly higher on weekdays compared to weekends (11,075 steps/day vs. 10,214 steps/day, respectively). We observed an inverse relationship between daily steps, particularly on weekdays, and adiposity parameters, notably among those who achieved over 12,000 steps/day and met the daily step recommendations at least 40% of the days, although this was only found in boys.

Previous research suggests that structured environments, such as school days, are associated with higher levels of PA due to the presence of scheduled physical education classes, recess periods, and after-school activities [37]. Our data are consistent with this, as they show that children tend to accumulate a greater number of daily steps on weekdays and that most (65%) of the children exhibit a stable pattern of activity between weekdays and weekends. However, it is also interesting that most children change towards a better category on the weekend (48 + 7 + 31/338 = 25.4%) than on the weekdays (10 + 0 + 22/338 = 9.5%). This consistency in PA levels may be attributed to individual behavioural patterns and routines, self-efficacy, and previous PA [38]. Furthermore, parental and peer support, as well as participation in organised sports, play crucial roles in maintaining PA levels throughout the week [39, 40]. Importantly, although the school environment favours high levels of PA during the week, the significant decrease in PA at weekends could promote sedentary behaviours [41] and negatively influence adiposity in the long term. It is also possible that the reduction in PA observed on weekends is partially due to increased sleep duration, which may act as a protective factor for adiposity [42, 43]. However, existing evidence suggests that PA remains a more robust predictor of adiposity in children and adolescents than sleep patterns or weekend catch-up sleep [44]. Therefore, we suggest that future interventions should focus on specific strategies to increase PA at weekends, involving both the family and the community.

A systematic review conducted by Miguel et al. [18], including 36 studies that assessed daily steps for 2–8 days, estimated negative associations between pedometer-measured PA and adiposity (as measured by BMI, WC, skinfold thickness, or bioelectrical impedance analysis), although data from included studies showed high heterogeneity and were insufficient to assess the dose–response relationship between PA and adiposity. Our study evidenced a clear dose–response between daily steps and adiposity parameters at a 30-week follow-up of daily steps. Nevertheless, only daily steps on weekdays are associated with a lower BMI among children who accumulated > 12,000 steps/day. This discrepancy may be because BF% and WC provide a more accurate measure of adiposity than BMI, which appears to vary according to the degree of fat mass and fat-free mass, especially in children and adolescents, because their weight, height, and body composition change during growth [45]. Furthermore, the most predictive PA indicator of adiposity was the number of steps taken per day during the school week. This may be because, as noted above, it represents the greatest proportion of the PA that children undertake during the week, and this structured weekday environment is beneficial in regulating obesogenic behaviours in children [41, 46].

Adherence to daily step recommendations also appears to play a crucial role in maintaining healthier body composition. Several studies [13, 47] observed that children who did not meet the recommended daily steps were more likely to be classified as having overweight or obesity and to have a higher WC compared to those who met the recommendations. Our data revealed that the percentage of days that the recommendations were met throughout the school year significantly influenced adiposity. Notably, a non-linear association was found, with the strongest effect observed when the recommendations were met on 20–40% of the days, after which the association stabilised. This phenomenon may be attributed to the fact that our sample met the recommendations on average only 32% of the days, and only 33% of the children met them more than 40% of the days measured. Alternatively, for children with very high compliance on certain days, there may be a trade-off with days of lower PA [48]. This suggests that the increase in steps on some days is compensated by a decrease on others, limiting the cumulative effect on adiposity. In addition, it is possible that children who meet the recommendations on more than 40% of days may add additional low-intensity steps, so that increased PA does not translate into proportionally higher energy expenditure [12], which could explain the plateau in adiposity protection. Future studies should investigate the role of step cadence.

Our results show discrepancies between sexes. In boys, an increase of 1000 steps/day, including weekdays, was associated with lower BMI, BF%, and WC, with the lowest levels observed at > 12,000 steps/day. In contrast, in girls, only those who accumulated > 12,000 steps/day on weekdays showed lower WC. Dollman et al. [49] observed a dose–response relationship between daily steps and BMI and WC across both sexes aged 5 to 12 years. However, when they performed the same analyses in adolescents aged 13 to 16 years, the ANCOVA models only demonstrated a dose–response for WC in boys. These discrepancies in the findings may be attributed to the disparate age groups under consideration, underscoring the necessity for further investigation across a broader age spectrum.

These recommendations (> 12,000 steps/day), which reflect a turning point at which significant benefits on adiposity parameters are observed, appear to be consistent with those proposed by Tudor-Locke et al. [12] for achieving 60 min of moderate-to-vigorous activity per day [11], where a minimum of 13,000–15,000 steps is indicated for boys and 11,000–12,000 for girls. This comparison highlights that, although there may be slight differences by sex, the thresholds we propose are consistent with previous guidelines and support the importance of promoting levels of physical activity that achieve these goals.

Finally, our findings support that measuring daily steps over an extended period of 30 weeks provides a more comprehensive and accurate representation of PA patterns in schoolchildren and their association with adiposity parameters. The extended monitoring period, which spans almost an entire academic year, encompasses the full spectrum of PA undertaken by children during their school year and mitigates the limitations of short-term assessments, such as the Hawthorne effect [19] by capturing more natural and consistent behaviours. Additionally, evidence indicates that children’s behaviour displays seasonal fluctuations [50], which are influenced by environmental changes [51]. Consequently, the number of daily steps taken by children varies throughout the year, both on weekends and weekdays [50]. This variability can impact the association with adiposity. In light of the aforementioned considerations, it seems pertinent to pose the following question: Has previous research underestimated the effect of PA on adiposity? Our methodology addressed this limitation by capturing these fluctuations, thereby providing a more accurate and reliable assessment of the relationship between PA and adiposity.

### Limitations

Some limitations must be recognised. First, a large proportion of participants (67.8%) were excluded due to missing data, potentially introducing selection bias. However, characteristics of included and excluded participants were similar, except for a higher percentage of girls in the final sample. This imbalance potentially compromises the representativeness of the sample and, by extension, the generalisability of the results, especially in the sex-stratified analysis. Second, regarding the external and cultural validity of the results, our data were collected from a convenience sample of Spanish children aged 9 to 13 years. We consider that future studies should focus on the replication of our findings in samples from different cultures and socioeconomic statuses. The results of the study need to be replicated in other populations to assess the consistency of the observed effects. Thirdly, it is important to note that the children were aware of their daily step counts, which may have influenced their behaviour, potentially leading to an increase in their activity levels. However, to mitigate this, we assessed daily steps over a longer period of time. Furthermore, the integration of both device-recorded and self-reported data may have introduced peer influence, thereby affecting the total number of steps. Fourth, the device used may not accurately capture non-ambulatory activities such as cycling or swimming. This could introduce a bias in the total estimate of PA. It is recommended that future studies consider the use of complementary devices to overcome this limitation. Fifth, we considered a valid day if the subjects wore the Xiaomi Mi Band 3 for more than 12 h. However, we acknowledge that if children removed the device during periods of PA, this could have resulted in an underestimation of the actual number of steps taken. Sixth, we included adherence to the Mediterranean diet and maternal education as confounders in the sensitivity analyses of ANCOVAs, but the sample size was reduced by half, which may have affected the statistical power. Furthermore, as dietary patterns may be a strong predictor of adiposity, we included adherence to the Mediterranean diet as a diet-related measured covariate; however, other food frequency tools should be used to more specifically estimate the daily energy expenditure. Finally, due to cultural restrictions and privacy constraints on physical examinations in school settings, information on sexual maturity was not included in the analysis due to the high non-response rate.

## Conclusions

Our results show that children undertake, on average, 10,823 steps/day (12,017 steps/day boys and 9848 steps/day girls), which is below the established recommendations. Children also tend to accumulate 861 more steps/day on weekdays compared to weekends over 30 weeks. We observed an inverse relationship between daily steps, especially on weekdays, and adiposity parameters, particularly among those who achieved over 12,000 steps/day and adhered to the daily step recommendations for at least 40% of the days. This relationship was more pronounced in the case of boys. In contrast, for girls, the association was primarily observed in WC. Sensitivity analysis confirmed that longer monitoring periods provided more accurate reflections of PA patterns.

## Supplementary Information

Additional file 1: Tables S1-S10 and Figures S1-S9. Table S1—STROBE Statement. Checklist of items that should be included in reports of longitudinal studies. Table S2—Characteristics of eMOVI participants that were included vs. excluded from the current analyses. Table S3—Characteristics of eMOVI participants that have at least one week of step data vs. eMOVI participants that have at least 18 of weeks of step data. Table S4—Mean difference between daily steps indicators by daily steps (complete week) categories and schools. Table S5—Cross-frequency table by categories of daily steps on weekdays and daily steps on weekend, by sex. Table S6—Analysis of covariance of adiposity indicators by categories of mean daily steps on complete week, weekdays, weekends, and percentage of days meeting daily step recommendations in girls. Table S7—Analysis of covariance of adiposity parameters by categories of mean daily steps on complete week, weekdays, weekends, and percentage of days meeting daily step recommendations in boys. Table S8—Multivariable mixed-effects linear regression model of 1,000 steps/day increment (for complete week, weekdays, and weekends) on adiposity parameters, by sex. Table S9—Analysis of covariance of adiposity indicators by categories of mean daily steps on complete week, weekdays, weekends, and percentage of days meeting daily step recommendations controlling age, sex, number of weeks with step data, adherence to mediterranean diet, and maternal education.Table S10—Analysis of covariance of adiposity parameters by categories of mean daily steps in a full week, on weekdays and on weekends, and percentage of days meeting daily step recommendations including subjects with at least 12 weeks of step data (n = 450). Fig. S1—Diagram flow of the study participants in the current study, from the original e-MOVI project. Fig. S2—Timetable for data collection. Fig. S3—Directed acyclic graph for the causal structure of the relationship between daily steps indicators and body mass index, percentage of body fat, and waist circumference. Fig. S4—Mean differences in body mass index, percentage of body fat, and waist circumference according to categories of mean daily steps in a complete week, on weekdays, and on weekend, and percentage of days meeting daily step recommendations, controlling for age, sex, and number of weeks with step data. The line indicates significant differences in the means (p < 0.05).Fig. S5—Restricted cubic splines with 95% confidence interval for the association of daily steps and percentage of days meeting daily step recommendations with body mass index (kg/m2) by sex, controlling for age and number of weeks with step data. Fig. S6—Restricted cubic splines with 95% confidence interval for the association of daily steps and percentage of days meeting daily step recommendations with percentage of body fat by sex, controlling for age and number of weeks with step data.Fig. S7—Restricted cubic splines with 95% confidence interval for the association of daily steps and percentage of days meeting daily step recommendations with waist circumference (cm) by sex, controlling for age and number of weeks with step data. Fig. S8—LOESS regression with 95% confidence interval for partial correlation coefficients (r) of daily steps during the complete week with adiposity parameters, controlling for age and sex by number of weeks of follow-up accumulated (1 to 24 weeks). Fig. S9—LOESS regression with 95% confidence interval for partial correlation coefficients (r) of daily steps during the complete week with adiposity parameters, controlling for age and sex by number of weeks with step data.

## Data Availability

The datasets used and/or analysed during the current study are available from the corresponding author on reasonable request.

## Abbreviations

ANCOVA: Analysis of covariance
BF%: Body fat percentage
BMI: Body mass index
CI: Confidence interval
DAG: Directed acyclic graph
LOESS: Locally estimated scatterplot smoothing
PA: Physical activity
WC: Waist circumference
