# Supplementary material for: Device-measured physical activity and adiposity in schoolchildren: a 30-week follow-up study
Source: BMC Med. 2025 Jun 2;23:329. doi: 10.1186/s12916-025-04161-4 (PMC12131665; doi:10.1186/s12916-025-04161-4)
Supplement: Supplementary file 1 — Additional file 1: Tables S1-S10 and Figures S1-S9. Table S1—STROBE Statement. Checklist of items that should be included in reports of longitudinal studies. Table S2—Characteristics of eMOVI participants that were included vs. excluded from the current analyses. Table S3—Characteristics of eMOVI participants that have at least one week of step data vs. eMOVI participants that have at least 18 of weeks of step data. Table S4—Mean difference between daily steps indicators by daily steps (complete week) categories and schools. Table S5—Cross-frequency table by categories of daily steps on weekdays and daily steps on weekend, by sex. Table S6—Analysis of covariance of adiposity indicators by categories of mean daily steps on complete week, weekdays, weekends, and percentage of days meeting daily step recommendations in girls. Table S7—Analysis of covariance of adiposity parameters by categories of mean daily steps on complete week, weekdays, weekends, and percentage of days meeting daily step recommendations in boys. Table S8—Multivariable mixed-effects linear regression model of 1,000 steps/day increment (for complete week, weekdays, and weekends) on adiposity parameters, by sex. Table S9—Analysis of covariance of adiposity indicators by categories of mean daily steps on complete week, weekdays, weekends, and percentage of days meeting daily step recommendations controlling age, sex, number of weeks with step data, adherence to mediterranean diet, and maternal education.Table S10—Analysis of covariance of adiposity parameters by categories of mean daily steps in a full week, on weekdays and on weekends, and percentage of days meeting daily step recommendations including subjects with at least 12 weeks of step data (n = 450). Fig. S1—Diagram flow of the study participants in the current study, from the original e-MOVI project. Fig. S2—Timetable for data collection. Fig. S3—Directed acyclic graph for the causal structure of the relationship between daily steps indicator [file 12916_2025_4161_MOESM1_ESM.docx]

**Additional file 1**

**Device-measured physical activity and adiposity in schoolchildren: a 30-week follow-up study**

**Short title:** Daily steps and adiposity in children

Eva Rodríguez-Gutiérrez, Msc ^a,b^, Vicente Martínez-Vizcaíno, PhD^a,c^*, Irene Sequí-Domínguez, PhD ^a,b^, Sergio Núñez de Arenas-Arroyo, PhD ^a,b^, Pontus Henriksson, PhD ^d^, Ángel Herraiz-Adillo, PhD ^d^, Ana Torres-Costoso, PhD ^a,e^

^a^ Health and Social Research Center, Universidad de Castilla-La Mancha, Cuenca, Spain

^b^ Research Network on Chronicity, Primary Care and Health Promotion (RICAPPS), Cuenca, Spain

^c^ Facultad de Ciencias de la Salud, Universidad Autónoma de Chile, Talca, Chile

^d^ Department of Health, Medicine and Caring Sciences, Linköping University, Linköping, Sweden

^e^ Facultad de Fisioterapia y Enfermería, Universidad de Castilla-La Mancha,

Toledo, Spain

***** **Corresponding author:** Vicente Martínez-Vizcaíno, Calle Santa Teresa de Jornet s/n, Cuenca, Spain. T.: +34 969 179 100 Ext: 4683. E-mail: [vicente.martinez@uclm.es](mailto:vicente.martinez@uclm.es)

[**Table S1.** STROBE Statement. Checklist of items that should be included in reports of longitudinal studies. 4](#_Toc198648545)

[**Table S2.** Characteristics of eMOVI participants that were included vs. excluded from the current analyses. 8](#_Toc198648546)

[**Table S3.** Characteristics of eMOVI participants that have at least one week of step data vs. eMOVI participants that have at least 18 of weeks of step data. 9](#_Toc198648547)

[**Table S4.** Mean difference between daily steps indicators by daily steps (complete week) categories and schools. 10](#_Toc198648548)

[**Table S5.** Cross-frequency table by categories of daily steps on weekdays and daily steps on weekend, by sex. 11](#_Toc198648549)

[**Table S6.** Analysis of covariance of adiposity indicators by categories of mean daily steps on complete week, weekdays, weekends, and percentage of days meeting daily step recommendations in girls. 12](#_Toc198648550)

[**Table S7.** Analysis of covariance of adiposity parameters by categories of mean daily steps on complete week, weekdays, weekends, and percentage of days meeting daily step recommendations in boys. 13](#_Toc198648551)

[**Table S8.** Multivariable mixed-effects linear regression model of 1,000 steps/day increment (for complete week, weekdays, and weekends) on adiposity parameters, by sex. 14](#_Toc198648552)

[**Table S9.** Analysis of covariance of adiposity indicators by categories of mean daily steps on complete week, weekdays, weekends, and percentage of days meeting daily step recommendations controlling age, sex, number of weeks with step data, adherence to mediterranean diet, and maternal education. 15](#_Toc198648553)

[**Table S10.** Analysis of covariance of adiposity parameters by categories of mean daily steps in a full week, on weekdays and on weekends, and percentage of days meeting daily step recommendations including subjects with at least 12 weeks of step data (*n = 450*). 16](#_Toc198648554)

[**Figure. S1.** Diagram flow of the study participants in the current study, from the original e-MOVI project. 17](#_Toc198648555)

[**Figure S2.** Timetable for data collection. 18](#_Toc198648556)

[**Figure S3.** Directed acyclic graph for the causal structure of the relationship between daily steps indicators and body mass index, percentage of body fat, and waist circumference. 19](#_Toc198648557)

[**Figure S4.** Mean differences in body mass index, percentage of body fat, and waist circumference according to categories of mean daily steps in a complete week, on weekdays, and on weekend, and percentage of days meeting daily step recommendations, controlling for age, sex, and number of weeks with step data. The line indicates significant differences in the means (p < 0.05). 20](#_Toc198648558)

[**Figure S5.** Restricted cubic splines with 95% confidence interval for the association of daily steps and percentage of days meeting daily step recommendations with body mass index (kg/m2) by sex, controlling for age and number of weeks with step data. 21](#_Toc198648559)

[**Figure S6.** Restricted cubic splines with 95% confidence interval for the association of daily steps and percentage of days meeting daily step recommendations with percentage of body fat by sex, controlling for age and number of weeks with step data. 22](#_Toc198648560)

[**Figure S7.** Restricted cubic splines with 95% confidence interval for the association of daily steps and percentage of days meeting daily step recommendations with waist circumference (cm) by sex, controlling for age and number of weeks with step data. 23](#_Toc198648561)

[**Figure S8.** LOESS regression with 95% confidence interval for partial correlation coefficients (r) of daily steps during the complete week with adiposity parameters, controlling for age and sex **by number of weeks of follow-up accumulated (1 to 24 weeks).** 24](#_Toc198648562)

[**Figure S9.** LOESS regression with 95% confidence interval for partial correlation coefficients (r) of daily steps during the complete week with adiposity parameters, controlling for age and sex **by number of weeks with step data.** 25](#_Toc198648563)

# **Table S1.** STROBE Statement. Checklist of items that should be included in reports of longitudinal studies.

|  | Item No | Recommendation | Location in the article |
| --- | --- | --- | --- |
| **Title and abstract** | 1 | (*a*) Indicate the study’s design with a commonly used term in the title or the abstract | Page 1, Line 1-2 |
|  |  | (*b*) Provide in the abstract an informative and balanced summary of what was done and what was found | Page 3 |
| Introduction | | | |
| Background/rationale | 2 | Explain the scientific background and rationale for the investigation being reported | 1. Introduction section: paragraph 1-4 |
| Objectives | 3 | State specific objectives, including any prespecified hypotheses | Introduction section: paragraph 5 |
| Methods | | | |
| Study design | 4 | Present key elements of study design early in the paper | 2.1. Study design and participants section: paragraph 1 |
| Setting | 5 | Describe the setting, locations, and relevant dates, including periods of recruitment, exposure, follow-up, and data collection | 2.1. Study design and participants section: paragraph 2  2.1. Study variables section, paragraph 1  Figure S2 |
| Participants | 6 | (*a*) Give the eligibility criteria, and the sources and methods of selection of participants | 2.1. Study design and participants section: paragraph 2 |
| Variables | 7 | Clearly define all outcomes, exposures, predictors, potential confounders, and effect modifiers. Give diagnostic criteria, if applicable | 2.1. Study variables sections: 2.2.1. Exposures: Daily steps, 2.2.2. Outcomes, 2.2.3. Covariates |
| Data sources/ measurement | 8 | For each variable of interest, give sources of data and details of methods of assessment (measurement). Describe comparability of assessment methods if there is more than one group | 2.1. Study variables sections |
| Bias | 9 | Describe any efforts to address potential sources of bias | 2.2.3. Covariates section |
| Study size | 10 | Explain how the study size was arrived at | 2.1. Study design and participants section: paragraph 2  Figure S1 |
| Quantitative variables | 11 | Explain how quantitative variables were handled in the analyses. If applicable, describe which groupings were chosen and why | 2.3. Statistical analysis section: paragraph 1 |
| Statistical methods | 12 | (*a*) Describe all statistical methods, including those used to control for confounding | 2.3. Statistical analysis section |
|  |  | (*b*) Describe any methods used to examine subgroups and interactions | 2.3. Statistical analysis section: paragraph 4 |
|  |  | (*c*) Explain how missing data were addressed | 2.1. Study design and participants section: paragraph 2  2.3. Statistical analysis section: paragraphs 1 and 5  Table S2, Table S3 and Table S10 |
|  |  | (*d*) If applicable, describe analytical methods taking account of sampling strategy | NA |
|  |  | (*e*) Describe any sensitivity analyses | 2.3. Statistical analysis section: paragraph 5 |
| Results | | | |
| Participants | 13 | (a) Report numbers of individuals at each stage of study—eg numbers potentially eligible, examined for eligibility, confirmed eligible, included in the study, completing follow-up, and analysed | 2.1. Study design and participants section: paragraph 2  Figure S1 |
|  |  | (b) Give reasons for non-participation at each stage | 2.1. Study design and participants section: paragraph 2  Figure S1 |
|  |  | (c) Consider use of a flow diagram | Figure S1 |
| Descriptive data | 14 | (a) Give characteristics of study participants (eg demographic, clinical, social) and information on exposures and potential confounders | 3.1. Descriptive data section  3.2. Differences in daily steps on weekdays and weekends section  Table 1, Table 2 and Figure S3 |
|  |  | (b) Indicate number of participants with missing data for each variable of interest | 2.1. Study design and participants section: paragraph 2  Figure S1 |
| Outcome data | 15 | Report numbers of outcome events or summary measures | Table 1 |
| Main results | 16 | (*a*) Give unadjusted estimates and, if applicable, confounder-adjusted estimates and their precision (eg, 95% confidence interval). Make clear which confounders were adjusted for and why they were included | 3.3. Association of daily steps indicators with adiposity parameters section: paragraph 2 and 3.  Table 2 and Table and S8  Figure 1-3, Figure S4 |
|  |  | (*b*) Report category boundaries when continuous variables were categorized | 3.2. Differences in daily steps on weekdays and weekends section  3.3. Association of daily steps indicators with adiposity parameters: paragraph 2  Table 2 and Table S5 Figure S4 |
|  |  | (*c*) If relevant, consider translating estimates of relative risk into absolute risk for a meaningful time period | NA |
| Other analyses | 17 | Report other analyses done—eg analyses of subgroups and interactions, and sensitivity analyses | 3.2. Differences in daily steps on weekdays and weekends section  3.3. Association of daily steps indicators with adiposity parameters  Tables S5, S6, S7, S9, and S10  Figure S5-S9 |
| Discussion | | | |
| Key results | 18 | Summarise key results with reference to study objectives | 4. Discussion section: paragraph 1 |
| Limitations | 19 | Discuss limitations of the study, taking into account sources of potential bias or imprecision. Discuss both direction and magnitude of any potential bias | 4. Discussion section: paragraphs 8 |
| Interpretation | 20 | Give a cautious overall interpretation of results considering objectives, limitations, multiplicity of analyses, results from similar studies, and other relevant evidence | Discussion section: paragraphs 2-7 |
| Generalisability | 21 | Discuss the generalisability (external validity) of the study results | 5. Conclusion section |
| Other information | | | |
| Funding | 22 | Give the source of funding and the role of the funders for the present study and, if applicable, for the original study on which the present article is based | Funding section |

# **Table S2.** Characteristics of eMOVI participants that were included vs. excluded from the current analyses.

|  | **Included**  (n = 338) | **Excluded^a^**  (n = 405) | **p-value** |
| --- | --- | --- | --- |
| Age (years) | 11.04 (10.94, 11.14) | 10.97 (10.88, 11.06) | 0.349 |
| Girls (%) | 55.03 | 44.69 | **0.005** |
| Height (cm) | 147.10 (146.13, 148.07) | 145.79 (144.93, 146.65) | 0.052 |
| Weight (kg) | 41.68 (40.55, 42.81) | 41.08 (40.01, 42.15) | 0.459 |
| Body mass index (kg/m^2^) | 19.00 (18.64, 19.36) | 19.07 (18.71, 19.43) | 0.781 |
| Body fat (%) | 24.76 (24.09, 25.43) | 24.41 (23.76, 25.06) | 0.466 |
| Waist circumference (cm) | 64.75 (63.74, 65.76) | 64.94 (63.98, 65.90) | 0.795 |
| Data are presented by mean and 95% confidence interval. ^a^ Children who consented to participate in eMOVI project but were excluded due to missing data on mean daily steps (complete week) (n = 389), mean daily weekdays steps (n = 381), and mean daily weekend steps (n = 405). | | | |

# **Table S3.** Characteristics of eMOVI participants that have at least one week of step data vs. eMOVI participants that have at least 18 of weeks of step data.

|  | **≥ 1 week and < 18 weeks** (n = 318) | **≥ 18 week** (n = 338) | **p-value** |
| --- | --- | --- | --- |
| Age (years) | 10.98 (10.87, 11.92) | 11.04 (10.94, 11.14) | 0.524 |
| Girls (%) | 55.03 | 45.91 | **0.020** |
| Height (cm) | 146.11 (145.14, 147.08) | 147.10 (146.13, 148.07) | 0.152 |
| Weight (kg) | 41.25 (40.01, 42.48) | 41.68 (40.55, 42.81) | 0.609 |
| Mean daily steps (complete week) | 11,033 (10,346, 12,520) | 10,823  (10,500, 11,146) | 0.231 |
| Mean daily weekdays steps | 11,284  (10,856, 11,713) | 11,075  (10,750, 11,392) | 0.208 |
| Mean daily weekend steps | 10,544  (10,093, 10,994) | 10,214  (9,860, 10,569) | 0.259 |
| Body mass index (kg/m^2^) | 18.83 (18.41, 19.26) | 19.00 (18.64, 19.36) | 0.905 |
| Body fat (%) | 24.33 (23.57, 25.09) | 24.76 (24.09, 25.43) | 0.666 |
| Waist circumference (cm) | 65.55 (64.38, 66.72) | 64.75 (63.74, 65.76) | 0.892 |
| Data are presented by mean and 95% confidence interval. | | | |

# **Table S4.** Mean difference between daily steps indicators by daily steps (complete week) categories and schools.

|  | | Daily steps (complete week) | Daily weekdays steps | Daily weekends steps | Mean difference between daily weekdays steps and daily weekend steps |
| --- | --- | --- | --- | --- | --- |
| **Daily steps (complete week) categories** | Low <9,000  (n = 105) | 7,595 (7,304, 7,886) | 7,772 (7,539, 8,005) | 6,867 (6,558, 7,176) | 905 (654, 1,155)** |
|  | Medium 9,000-12,000  (n = 119) | 10,777 (10,504, 11,050) | 10,790 (10,617, 10,963) | 9,938 (9,636, 10,240) | 852 ± (516, 1,188)** |
|  | High >12,000  (n = 114) | 14,789 (14,335, 15,242) | 14,386 (14,026, 14,745) | 13,585 (13,148, 14,022) | 801 (423, 1,178)** |
| **Schools** | School 1  (n = 46) | 11,403 (10,511, 12,294) | 11,588 (10,695, 12,480) | 10,677 (9,740, 11,613) | 911 (582, 1,239)** |
|  | School 2  (n = 42) | 10,549 (9,690, 11,407) | 10,875 (10,008, 11,741) | 9,716 (8,779, 10,652) | 1,159 (635, 1,682)** |
|  | School 3  (n = 55) | 10,520 (9,634, 11,405) | 10,672 (9,828, 11,515) | 10,256 (9,267, 11,244) | 416 (51, 883) |
|  | School 4  (n = 81) | 10,931 (10,273, 11,588) | 11,153 (10,523, 11,782) | 10,377 (9,634, 11,119) | 776 (393, 1,158)** |
|  | School 5  (n = 59) | 11,321 (10,519, 12,122) | 11,557 (10,753, 12,360) | 10,925 (10,081, 11,768) | 632 (87, 1,176)* |
|  | School 6  (n = 55) | 10,157 (9,475, 10,838) | 10,568 (9,880, 11,255) | 9,163 (8,420, 9,905) | 1,405 (950, 1,859)** |
| Data are presented by mean and 95% confidence interval.  * p < 0.05 ** p < 0.001 | | | | | |

# **Table S5.** Cross-frequency table by categories of daily steps on weekdays and daily steps on weekend, by sex.

| **Total sample** | | | | | |
| --- | --- | --- | --- | --- | --- |
|  | | Daily weekdays steps | | |  |
|  | Categories | < 9.000 steps/day | 9.000-12.000 steps/day | > 12.000 steps/day | n total |
| Daily weekend steps | < 9.000 steps/day | 77 | 10 | 0 | 87 |
|  | 9.000-12.000 steps/day | 48 | 62 | 22 | 132 |
|  | > 12.000 steps/day | 7 | 31 | 81 | 119 |
|  | n total | 132 | 103 | 103 | 338 |
| **Girls** | | | | | |
|  | | Daily weekdays steps | | |  |
|  | Categories | < 9.000 steps/day | 9.000-12.000 steps/day | > 12.000 steps/day | n total |
| Daily weekend steps | < 9.000 steps/day | 61 | 6 | 0 | 67 |
|  | 9.000-12.000 steps/day | 29 | 38 | 16 | 83 |
|  | > 12.000 steps/day | 2 | 8 | 26 | 36 |
|  | n total | 92 | 52 | 42 | 186 |
| **Boys** | | | | | |
|  | | Daily weekdays steps | | |  |
|  | Categories | < 9.000 steps/day | 9.000-12.000 steps/day | > 12.000 steps/day | n total |
| Daily weekend steps | < 9.000 steps/day | 16 | 4 | 0 | 20 |
|  | 9.000-12.000 steps/day | 19 | 24 | 6 | 49 |
|  | > 12.000 steps/day | 5 | 23 | 55 | 83 |
|  | n total | 40 | 51 | 61 | 152 |
| Total sample: observed proportionate agreement: 65.01%; weighted Cohen’s kappa: 0.481  Girls: observed proportionate agreement: 67.20%; weighted Cohen’s kappa: 0.498  Boys: observed proportionate agreement: 62.50%; weighted Cohen’s kappa: 0.412 | | | | | |

# **Table S6.** Analysis of covariance of adiposity indicators by categories of mean daily steps on complete week, weekdays, weekends, and percentage of days meeting daily step recommendations in girls.

|  | | n | Body mass index | | % Body fat | | Waist circumference | |
| --- | --- | --- | --- | --- | --- | --- | --- | --- |
|  |  |  | M0 | M1 | M0 | M1 | M0 | M1 |
| **Mean daily steps (complete week) categories** | Low (L) <9,000 | 80 | 19.37  (18.61, 20.13) | 19.33  (18.56, 20.10) | 27.06  (25.91, 28.21) | 27.02  (25.86, 28.18) | 65.77  (63.61, 67.93) | 65.61  (63.46, 67.76) |
|  | Medium (M) 9,000-12,000 | 69 | 19.12  (18.28, 19.96) | 19.23  (18.38, 20.08) | 26.55  (25.32, 27.78) | 26.58  (25.34, 27.82) | 64.24  (62.34, 66.14) | 64.70  (62.80, 66.60) |
|  | High (H) >12,000 | 37 | 18.61  (17.48, 19.74) | 18.51  (17.38, 19.64) | 25.66  (24.00, 27.32) | 25.68  (23.99, 27.37) | 62.19  (59.18, 65.20) | 61.67  (58.68, 64.66) |
|  | p-value |  | 0.553 | 0.492 | 0.484 | 0.523 | 0.174 | 0.120 |
| **Mean daily weekdays steps** | Low (L) <9,000 | 67 | 19.48  (18.66, 20.30) | 19.44  (18.62, 20.26) | 27.05  (25.78, 28.32) | 27.02  (25.75, 28.29) | 66.07  (63.82, 68.32) | 65.88  (63.64, 68.12) ^H^ |
|  | Medium (M) 9,000-12,000 | 83 | 19.19  (18.48, 19.90) | 19.26  (18.55, 19.97) | 26.86  (25.64, 28.08) | 26.89  (25.66, 28.12) | 64.55  (62.43, 66.67) | 64.91  (62.79, 67.03) |
|  | High (H) >12,000 | 36 | 18.33  (17.29, 19.37) | 18.23  (17.19, 19.27) | 25.09  (23.52, 26.66) | 25.08  (23.50, 26.66) | 61.41  (58.84, 63.98) | 60.91  (58.35, 63.47) ^L^ |
|  | p-value |  | 0.281 | 0.226 | 0.230 | 0.234 | 0.067 | **0.038** |
| **Mean daily weekend steps** | Low (L) <9,000 | 92 | 19.52  (18.81, 20.23) | 19.52  (18.79, 20.25) | 27.43  (26.29, 28.57) | 27.44  (26.28, 28.60) | 65.88  (63.61, 68.15) | 65.91  (63.64, 68.18) |
|  | Medium (M) 9,000-12,000 | 52 | 18.65  (17.66, 19.64) | 18.70  (17.71, 19.69) | 25.8  (24.05, 27.55) | 25.8  (24.05, 27.55) | 63.03  (60.30, 65.76) | 63.26  (60.52, 66.00) |
|  | High (H) >12,000 | 42 | 18.83  (17.57, 20.09) | 18.77 (17.50, 20.04) | 25.7  (23.63, 27.77) | 25.68  (23.57, 27.79) | 63.19  (60.32, 66.06) | 62.83  (59.93, 65.73) |
|  | p-value |  | 0.308 | 0.320 | 0.141 | 0.147 | 0.148 | 0.132 |
| **% Days meeting daily step recommendations** | Low (L) <20% | 76 | 19.66  (18.86, 20.46) ^M^ | 19.59  (18.77, 20.41) ^M^ | 27.55  (26.26, 28.84) | 27.52  (26.10, 28.94) | 66.42  (64.19, 68.65) ^M^ | 66.31  (63.95, 68.67) ^M^ |
|  | Medium (M) 20-40% | 51 | 18.06  (17.94, 20.18) ^L^ | 18.08  (17.96, 20.20) ^L^ | 25.37  (23.94, 26.80) | 25.37  (23.94, 26.80) | 61.8  (59.09, 64.51) ^L^ | 61.89  (59.20, 64.58) ^L^ |
|  | High (H) >40% | 57 | 19.39  (18.56, 20.22) | 19.46  (18.58, 20.34) | 26.44  (25.12, 27.76) | 26.48  (25.10, 27.86) | 64.37  (62.00, 66.74) | 64.44  (61.92, 66.96) |
|  | p-value |  | **0.031** | **0.038** | 0.105 | 0.127 | **0.029** | **0.040** |
| Data are presented as mean and 95% confidence interval. The values in bold indicate statistical significance at p < 0.05. Model 0 (M0): unadjusted data analysis. Model 1 (M1): controlling for age, sex, and number of weeks with step data. Superscript letter indicates statistical significance (p < 0.05) between categories for post-hoc tests using the Bonferroni comparisons. | | | | | | | | |

# **Table S7.** Analysis of covariance of adiposity parameters by categories of mean daily steps on complete week, weekdays, weekends, and percentage of days meeting daily step recommendations in boys.

|  | | n | Body mass index | | % Body fat | | Waist circumference | |
| --- | --- | --- | --- | --- | --- | --- | --- | --- |
|  |  |  | M0 | M1 | M0 | M1 | M0 | M1 |
| **Mean daily steps (complete week) categories** | Low (L) <9,000 | 25 | 19.43  (18.29, 20.57) | 19.40  (18.26, 20.54) | 24.04  (21.77, 26.31) ^H^ | 24.39  (22.13, 26.65) ^H^ | 67.28  (63.36, 71.20) ^H^ | 67.12  (63.21, 71.03) ^H^ |
|  | Medium (M) 9,000-12,000 | 50 | 19.20  (18.34, 20.06) | 19.28  (18.42, 20.14) | 23.76  (22.06, 25.46) | 23.63  (21.95, 25.31) | 66.86  (64.23, 69.49) | 67.17  (64.54, 69.80) |
|  | High (H) >12,000 | 77 | 18.43  (17.66, 19.20) | 18.39  (17.62, 19.16) | 21.29  (19.77, 22.81) ^L^ | 21.26  (19.77, 22.75) ^L^ | 63.20  (61.00, 65.40) ^L^ | 63.05  (60.86, 65.24) ^L^ |
|  | p-value |  | 0.275 | 0.221 | **0.041** | **0.031** | **0.041** | **0.020** |
| **Mean daily weekdays steps** | Low (L) <9,000 | 20 | 20.09   (18.47, 21.71) | 20.04  (18.41, 21.67) | 24.46  (21.56, 27.36) | 25.00  (22.10, 27.90) ^H^ | 68.59  (64.60, 72.58) ^H^ | 68.22  (64.21, 72.23) |
|  | Medium (M) 9,000-12,000 | 49 | 19.00  (18.84, 19.16) | 19.15  (18.99, 19.31) | 23.81  (22.06, 25.56) | 23.67  (21.92, 25.42) | 66.54  (64.23, 69.49) | 67.09  (64.54, 69.80) |
|  | High (H) >12,000 | 83 | 18.45  (17.66, 19.24) | 18.38  (17.59, 19.17) | 21.35  (19.77, 22.93) | 21.30  (19.72, 22.88) ^L^ | 63.35  (61.00, 65.70) ^L^ | 63.13  (60.78, 65.48) ^L^ |
|  | p-value |  | 0.123 | 0.102 | **0.032** | **0.019** | **0.031** | **0.018** |
| **Mean daily weekend steps** | Low (L) <9,000 | 40 | 19.02  (18.81, 19.23) | 19.09 (18.88, 19.30) | 23.55  (21.25, 25.85) | 23.42  (21.12, 25.72) | 65.75  (63.61, 67.89) | 66.04  (63.90, 68.18) |
|  | Medium (M) 9,000-12,000 | 51 | 19.16  (18.66, 19.66) | 19.19  (18.69, 19.71) | 23.67  (22.05, 25.29) | 23.58  (21.96, 25.20) | 66.77  (64.23, 69.31) | 66.93  (64.39, 69.47) |
|  | High (H) >12,000 | 61 | 18.47  (17.57, 19.37) | 18.40  (17.50, 19.30) | 20.96  (19.77, 22.15) | 21.12  (19.93, 22.31) | 63.20  (61.00, 65.40) | 62.87  (60.67, 65.07) |
|  | p-value |  | 0.511 | 0.401 | **0.036** | 0.072 | 0.111 | 0.055 |
| **% Days meeting daily step recommendations** | Low (L) <20% | 41 | 19.43  (18.29, 20.57) | 19.45  (18.31, 20.59) | 24.07  (21.77, 26.37) ^H^ | 23.94  (21.64, 26.24) ^H^ | 67.7  (63.36, 72.04) ^H^ | 67.82  (63.48, 72.16) ^H^ |
|  | Medium (M) 20-40% | 52 | 19.10  (18.34, 19.86) | 19.08  (18.32, 19.84) | 23.28  (22.06, 25.50) | 23.32  (22.10, 25.54) | 65.26  (64.23, 69.31) | 65.18  (64.15, 69.21) |
|  | High (H) >40% | 59 | 18.22  (17.66, 18.78) | 18.22  (17.66, 18.78) | 20.87  (19.77, 22.15) ^L^ | 20.92  (19.82, 22.02) ^L^ | 63.1  (61.00, 65.20) ^L^ | 63.09  (60.99, 65.19) ^L^ |
|  | p-value |  | 0.155 | 0.156 | **0.025** | **0.030** | 0.052 | **0.044** |
| Data are presented as mean and 95% confidence interval. The values in bold indicate statistical significance at p < 0.05. Model 0 (M0): unadjusted data analysis. Model 1 (M1): controlling for age, sex, and number of weeks with step data. Superscript letter indicates statistical significance (p < 0.05) between categories for post-hoc tests using the Bonferroni comparisons. | | | | | | | | |

# **Table S8.** Multivariable mixed-effects linear regression model of 1,000 steps/day increment (for complete week, weekdays, and weekends) on adiposity parameters, by sex.

|  |  |  | Body mass index | | % Body fat | | Waist circumference | |
| --- | --- | --- | --- | --- | --- | --- | --- | --- |
| 1,000 steps/day increment |  |  | β ± SD | p-value | β ± SD | p-value | β ± SD | p-value |
| Complete week | Total sample | M_0_ | -0.13 ± 1.14 | **0.026** | -0.52 ± 1.98 | 0.051 | -0.41 ± 3.09 | **0.016** |
|  |  | M_1_ | -0.14 ± 1.19 | **0.029** | -0.33 ± 2.06 | **0.004** | -0.51 ± 3.26 | **0.004** |
|  | Girls | M_0_ | -0.12 ± 1.06 | 0.212 | -0.21 ± 2.16 | 0.203 | -0.46 ± 3.63 | 0.088 |
|  |  | M_1_ | -0.12 ± 1.33 | 0.228 | -0.20 ± 2.20 | 0.221 | -0.46 ± 3.60 | 0.084 |
|  | Boys | M_0_ | -0.16 ± 1.31 | 0.057 | -0.47 ± 1.95 | **0.004** | -0.57 ± 2.97 | **0.020** |
|  |  | M_1_ | -0.18 ± 1.07 | **0.043** | -0.48 ± 1.97 | **0.003** | -0.63 ± 3.00 | **0.011** |
| Weekday | Total sample | M_0_ | -0.15 ± 1.13 | **0.013** | -0.56 ± 2.00 | **<0.001** | -0.46 ± 3.15 | **0.008** |
|  |  | M_1_ | -0.17 ± 1.21 | **0.013** | -0.36 ± 2.12 | **0.002** | -0.59 ± 3.35 | **0.001** |
|  | Girls | M_0_ | -0.12 ± 1.34 | 0.217 | -0.20 ± 2.21 | 0.214 | -0.46 ± 3.69 | 0.095 |
|  |  | M_1_ | -0.12 ± 1.35 | 0.220 | -0.20 ± 2.23 | 0.226 | -0.47 ± 3.65 | 0.086 |
|  | Boys | M_0_ | -0.20 ± 1.08 | **0.022** | -0.53 ± 2.00 | **0.001** | -0.71 ± 3.05 | **0.005** |
|  |  | M_1_ | -0.22 ± 1.11 | **0.014** | -0.55 ± 2.05 | **0.001** | -0.78 ± 3.10 | **0.002** |
| Weekend | Total sample | M_0_ | -0.09 ± 1.00 | 0.093 | -0.37 ± 1.84 | **<0.001** | -0.29 ± 2.82 | 0.061 |
|  |  | M_1_ | -0.09 ± 1.04 | 0.102 | -0.23 ± 1.82 | **0.020** | -0.35 ± 2.87 | **0.028** |
|  | Girls | M_0_ | -0.12 ± 1.15 | 0.167 | -0.21 ± 1.89 | 0.144 | -0.38 ± 3.16 | 0.107 |
|  |  | M_1_ | -0.12 ± 1.16 | 0.156 | -0.21 ± 1.93 | 0.148 | -0.41 ± 3.18 | 0.080 |
|  | Boys | M_0_ | -0.08 ± 0.93 | 0.321 | -0.29 ± 1.74 | **0.046** | -0.31 ± 3.03 | 0.149 |
|  |  | M_1_ | -0.08 ± 0.95 | 0.317 | -0.26 ± 1.76 | 0.067 | -0.34 ± 2.67 | 0.124 |
| Data are presented as unstandardised beta coefficient (β) ± standard deviation. The values in bold indicate statistical significance at p < 0.05. Model 0 (M0): unadjusted data analysis. Model 1 (M1): controlling for age, sex (only total sample), and number of weeks with step data. | | | | | | | | |

# **Table S9.** Analysis of covariance of adiposity indicators by categories of mean daily steps on complete week, weekdays, weekends, and percentage of days meeting daily step recommendations controlling age, sex, number of weeks with step data, adherence to mediterranean diet, and maternal education.

|  | | n | Body mass index | | | % Body fat | | | | | | Waist circumference | | | |
| --- | --- | --- | --- | --- | --- | --- | --- | --- | --- | --- | --- | --- | --- | --- | --- |
|  |  |  | M0 | M1 | | | M0 | | | M1 | | M0 | | M1 | |
| **Mean daily steps (complete week) categories** | Low (L) <9,000 | 105 (M0)  55 (M1) | 19.39  (18.73, 20.04) | 19.47  (18.51, 20.42) | | | 26.35  (25.17, 27.53) ^H^ | | | 26.03  (24.31, 27.76) | 66.13  (64.32, 67.94) ^H^ | | | 66.85  (64.27, 69.43) |  |
|  | Medium (M) 9,000-12,000 | 119 (M0)  61 (M1) | 19.15  (18.54, 19.77) | 19.31  (18.43, 20.19) | | | 25.38  (24.27, 26.49) ^H^ | | | 25.09  (23.51, 26.66) | 65.34  (63.63, 67.04) | | | 66.02  (63.64, 68.40) |  |
|  | High (H) >12,000 | 114 (M0)  59 (M1) | 18.49  (17.86, 19.11) | 18.79  (17.87, 19.72) | | | 22.66  (21.52, 23.79) ^L,M^ | | | 24.20  (22.53, 25.88) | 62.88  (61.14, 64.61) ^L^ | | | 62.73  (60.23, 65.23) |  |
|  | p-value |  | 0.124 | 0.593 | | | **<0.001** | | | 0.351 | **0.028** | | | 0.066 |  |
| **Mean daily weekdays steps** | Low (L) <9,000 | 87 (M0)  43 (M1) | 19.62  (18.91, 20.34) ^H^ | 19.63  (18.55, 20.72) | | | 26.45  (25.16, 27.73) ^H^ | | | 26.03  (24.09, 27.96) | 66.66  (64.67, 68.64) ^H^ | | | 67.29  (64.37, 68.66) ^H^ |  |
|  | Medium (M) 9,000-12,000 | 132 (M0)  69 (M1) | 19.12  (19.54, 19.70) | 19.50  (18.67, 20.33) | | | 25.75  (24.70, 29.80) ^H^ | | | 25.65  (24.15, 27.14) | 65.28  (63.67, 66.90) | | | 66.43  (64.19, 68.66) |  |
|  | High (H) >12,000 | 119 (M0)  63 (M1) | 18.42  (17.81, 19.03) ^L^ | 18.53  (17.64, 19.43) | | | 22.44  (21.34, 23.54) ^L,M^ | | | 23.82  (22.20, 25.44) | 62.78  (61.08, 64.47) ^L^ | | | 62.35  (59.94, 64.77) ^L^ |  |
|  | p-value |  | **0.037** | 0.216 | | | **<0.001** | | | 0.170 | **0.010** | | | **0.020** |  |
| **Mean daily weekend steps** | Low (L) <9,000 | 132 (M0)  75 (M1) | 19.37  (18.79, 19.95) | 19.42  (18.62, 20.23) | | | 26.29  (25.23, 27.35) ^H^ | | | 25.89  (24.45, 27.34) | 65.84 (64.22, 67.46) | | | 66.92  (64.75, 69.09) |  |
|  | Medium (M) 9,000-12,000 | 103 (M0)  50 (M1) | 18.91  (18.24, 19.58) | 19.13  (18.16, 20.11) | | | 24.71  (23.51, 25.92) | | | 24.96  (23.22, 26.70) | 64.93 (63.08, 66.79) | | | 65.02  (62.38, 67.65) |  |
|  | High (H) >12,000 | 103 (M0)  50 (M1) | 18.62  (17.96, 19.28) | 18.89  (17.90, 19.87) | | | 22.87  (21.67, 24.06) ^L^ | | | 24.00  (22.23, 25.76) | 63.19 (61.37, 65.02) | | | 62.71  (60.07, 65.35) |  |
|  | p-value |  | 0.232 | 0.709 | | | **<0.001** | | | 0.269 | 0.102 | | | 0.058 |  |
| **% Days meeting daily step recommendations** | Low (L) <20% | 117 (M0)  47 (M1) | 19.58  (18.97, 20.19) | 19.66  (18.66, 20.67) | | | 26.36  (25.24, 27.48) ^M,H^ | | | 26.53  (24.72, 28.35) | 66.87  (65.17, 68.57) ^M,H^ | | | 67.95  (65.24, 70.66) ^H^ |  |
|  | Medium (M) 20-40% | 105 (M0)  79 (M1) | 18.58  (17.94, 19.22) | 19.16  (18.38, 19.94) | | | 24.32  (23.14, 25.50) ^L^ | | | 24.86  (23.48, 26.24) | 63.55  (61.76, 65.34) ^L^ | | | 65.10  (63.02, 67.19) |  |
|  | High (H) >40% | 116 (M0)  49 (M1) | 18.80  (18.19, 19.41) | 18.77  (17.79, 19.75) | | | 23.58  (22.45, 24.71) ^L^ | | | 24.07  (22.31, 25.83) | 63.73  (62.03, 65.43) ^L^ | | | 62.62  (60.00, 65.25) ^L^ |  |
|  | p-value |  | 0.070 | | 0.455 | | | **0.002** | 0.149 | | | **0.012** | **0.023** | | |
| Data are presented as mean and 95% confidence interval. The values in bold indicate statistical significance at p < 0.05. Model 0 (M0): unadjusted data analysis. Model 1 (M1): controlling for age, sex, number of weeks with step data, adherence to mediterranean diet, and maternal education. Superscript letter indicates statistical significance (p < 0.05) between categories for post-hoc tests using the Bonferroni comparisons. | | | | | | | | | | | | | | | |

# **Table S10.** Analysis of covariance of adiposity parameters by categories of mean daily steps in a full week, on weekdays and on weekends, and percentage of days meeting daily step recommendations including subjects with at least 12 weeks of step data (*n = 450*).

|  | | n | Body mass index | | % Body fat | | Waist circumference | |
| --- | --- | --- | --- | --- | --- | --- | --- | --- |
|  |  |  | M0 | M1 | M0 | M1 | M0 | M1 |
| **Mean daily steps (complete week) categories** | Low (L) <9,000 | 130 | 19.43  (18.84, 20.02) ^H^ | 19.32  (18.71, 19.93) | 26.47  (25.40, 27.54) ^H^ | 25.64  (24.57, 26.72) ^H^ | 66.05  (64.43, 67.67) ^H^ | 66.17  (64.53, 67.82) ^H^ |
|  | Medium (M) 9,000-12,000 | 142 | 19.08  (18.51, 19.64) | 19.16  (18.59, 19.72) | 25.03  (24.02, 26.05) ^H^ | 24.98  (23.99, 25.97) ^H^ | 65.20  (63.63, 66.75) | 65.53  (64.00, 67.06) ^H^ |
|  | High (H) >12,000 | 178 | 18.42  (17.91, 18.92) ^L^ | 18.43  (17.91, 18.95) | 22.67  (21.76, 23.58) ^L,M^ | 23.32  (22.40, 24.23) ^L,M^ | 62.87  (61.49, 64.25) ^L^ | 62.50  (61.11, 63.92) ^L,M^ |
|  | p-value |  | **0.032** | 0.065 | **<0.001** | **0.004** | **0.008** | **0.002** |
| **Mean daily weekdays steps** | Low (L) <9,000 | 118 | 19.61  (18.99, 20.23) ^H^ | 19.54  (18.91, 20.17) ^H^ | 26.50  (25.40, 27.60) ^H^ | 25.77  (24.66, 26.87) ^H^ | 66.57  (64.88, 68.25) ^H^ | 66.76  (65.05, 68.46) ^H^ |
|  | Medium (M) 9,000-12,000 | 160 | 19.18  (18.65, 19.71) | 19.26  (18.73, 19.79) ^H^ | 25.54  (24.59, 26.49) ^H^ | 25.36  (24.43, 26.29) ^H^ | 65.36  (63.91, 66.81) ^H^ | 65.78  (64.34, 67.21) ^H^ |
|  | High (H) >12,000 | 172 | 18.20  (17.69, 18.71) ^L^ | 18.17  (17.65, 18.70) ^L,M^ | 22.19  (21.27, 23.10) ^L,M^ | 22.86  (21.93, 23.79) ^L,M^ | 62.35  (60.95, 63.75) ^L,M^ | 61.83  (60.41, 63.25)  ^L,M^ |
|  | p-value |  | **0.001** | **0.002** | **<0.001** | **<0.001** | **<0.001** | **<0.001** |
| **Mean daily weekend steps** | Low (L) <9,000 | 163 | 19.30  (18.77, 19.83) | 19.27  (18.73, 19.80) | 26.05  (25.09, 27.00) ^H^ | 25.41  (24.47, 26.36) ^H^ | 65.66  (64.21, 67.11) ^H^ | 65.89  (64.44, 67.35) ^H^ |
|  | Medium (M) 9,000-12,000 | 139 | 18.93  (18.36, 19.51) | 19.01  (18.44, 19.58) | 24.72  (23.68, 25.75) ^H^ | 24.89  (23.89, 25.89) ^H^ | 64.90  (63.33, 66.47) | 65.09  (63.55, 66.64) |
|  | High (H) >12,000 | 148 | 18.48  (17.93, 19.04) | 18.45  (17.89, 19.01) | 22.64  (21.63, 23.64) ^L,M^ | 23.17  (22.18, 24.16) ^L,M^ | 62.92  (61.40, 64.44) ^L^ | 62.48  (60.96, 64.00) ^L^ |
|  | p-value |  | 0.113 | 0.119 | **<0.001** | **0.005** | **0.033** | **0.005** |
| **% Days meeting daily step recommendations** | Low (L) <20% | 115 | 19.71  (19.08, 20.34) ^H^ | 19.67  (19.05, 20.29) ^H^ | 26.39  (25.24, 27.53)^M,H^ | 26.04  (24.94, 27.14) ^H^ | 67.01  (65.30, 68.72) ^H^ | 67.03  (65.35, 68.72) ^H^ |
|  | Medium (M) 20-40% | 198 | 18.86  (18.39, 19.34) | 18.89  (18.42, 19.37) | 24.58  (23.71, 25.45) ^L,H^ | 24.58  (23.75, 25.41) | 64.63  (63.32, 65.93) | 64.73  (63.44, 66.01)  ^H^ |
|  | High (H) >40% | 137 | 18.33  (17.76, 18.91) ^L^ | 18.33  (17.75, 18.90) ^L^ | 22.85  (21.80, 23.90) ^L,M^ | 23.14  (22.13, 24.15) ^L^ | 62.30  (60.73, 63.86) ^L^ | 62.13  (60.58, 63.68) ^L,M^ |
|  | p-value |  | **0.006** | **0.008** | **0.038** | **<0.001** | **<0.001** | **<0.001** |
| Data are presented as mean and 95% confidence interval. The values in bold indicate statistical significance at p < 0.05. Model 0 (M0): unadjusted data analysis. Model 1 (M1): controlling for age, sex, and number of weeks with step data. Superscript letter indicates statistical significance (p < 0.05) between categories for post-hoc tests using the Bonferroni comparisons. | | | | | | | | |

Respondents:

745

Invited for participation:

1,049

Non-respondents:

304 actively refused or did not return the written approval.

Excluded: 407

- 391 had missing data on mean daily steps (complete week).
- 403 had missing data on mean daily weekend steps.
- 383 had missing data on mean daily weekdays steps.

Population for analyses:

338

# **Figure. S1.** Diagram flow of the study participants in the current study, from the original e-MOVI project.


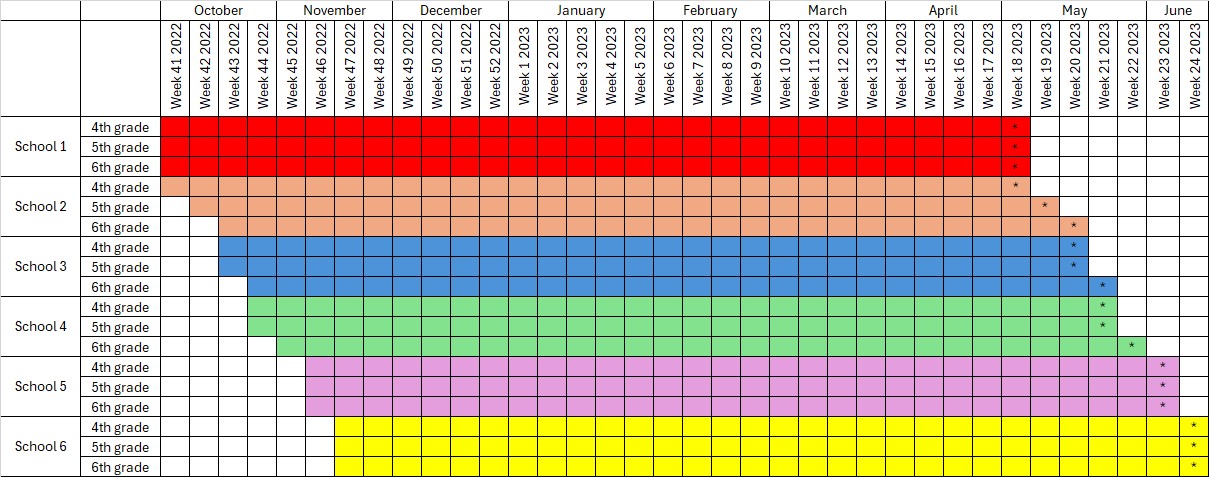


Coloured cells show the follow-up of the daily step data collection.

* Indicates the date of collection of adiposity parameters.

# **Figure S2.** Timetable for data collection.

1. Original directed acyclic graph


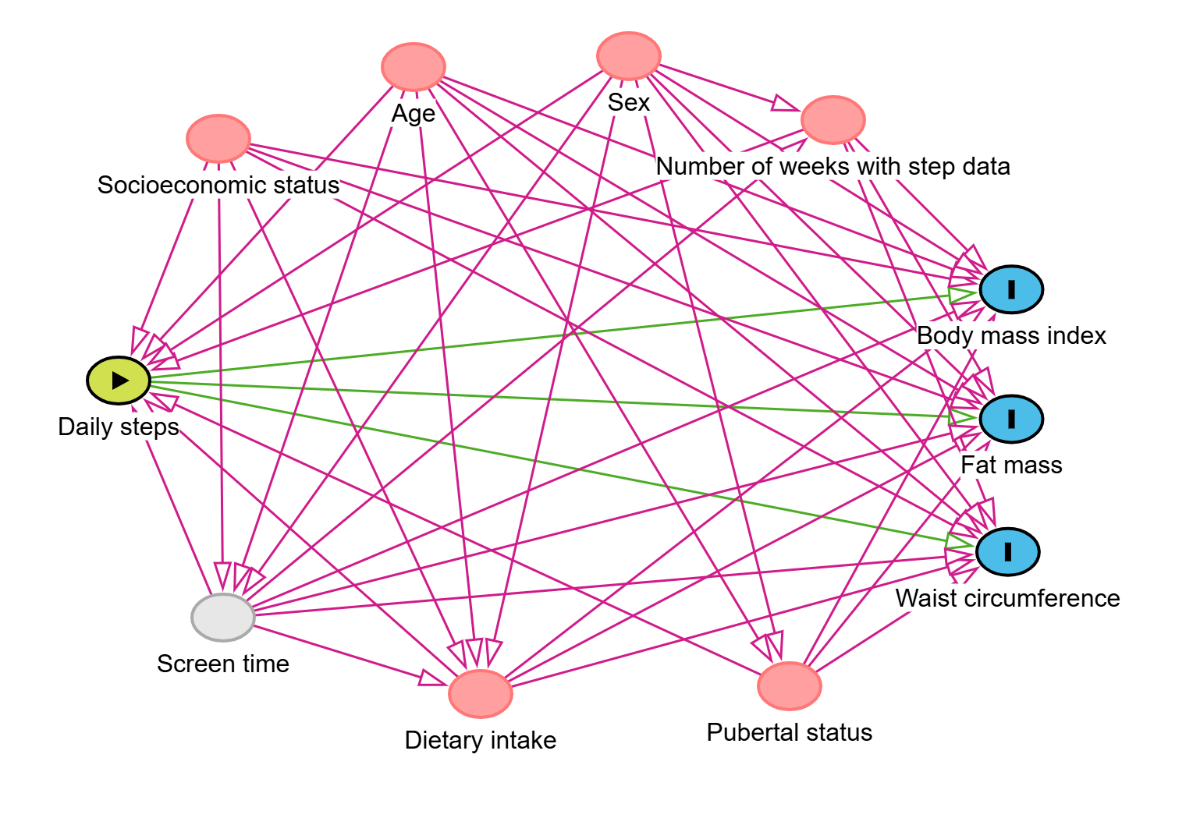


1. Directed acyclic graph after adjustment for the covariates available


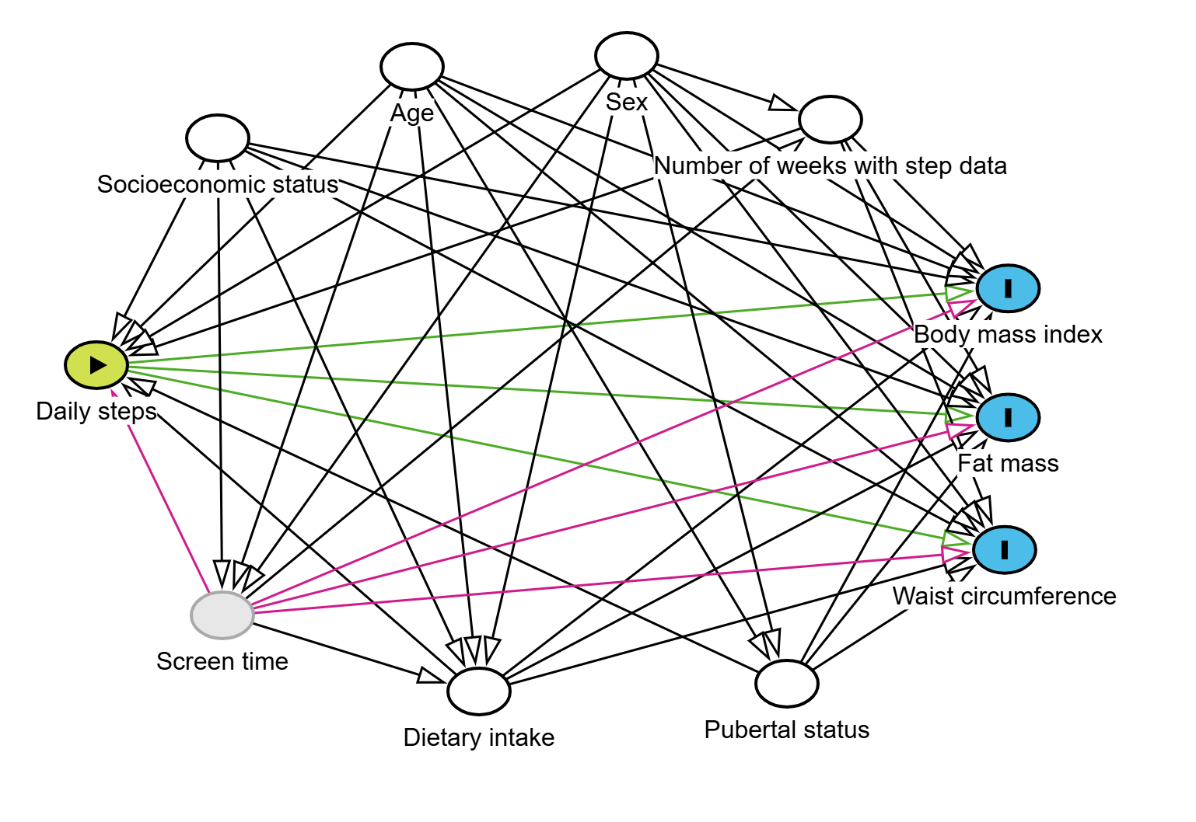


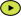
 Exposure,
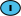
 Outcome,
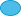
 Ancestor of outcome,
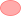
 Ancestor of exposure and outcome, …. Unavailable variable,
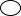
Adjusted variable,
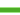
 Causal path,
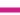
 Biasing path, Biasing path closed

# **Figure S3.** Directed acyclic graph for the causal structure of the relationship between daily steps indicators and body mass index, percentage of body fat, and waist circumference.

***
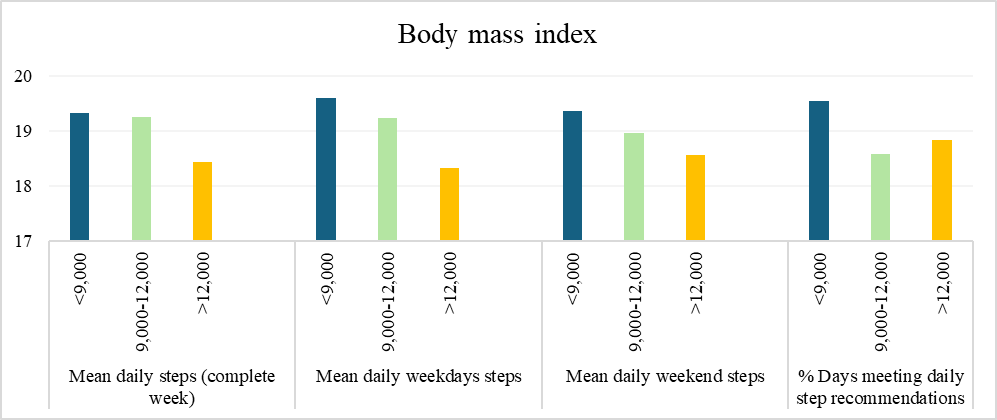
S***

***
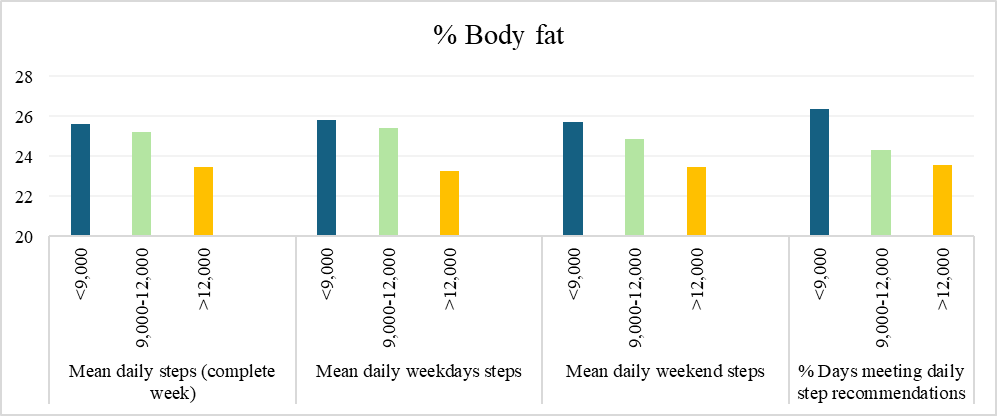
***

***
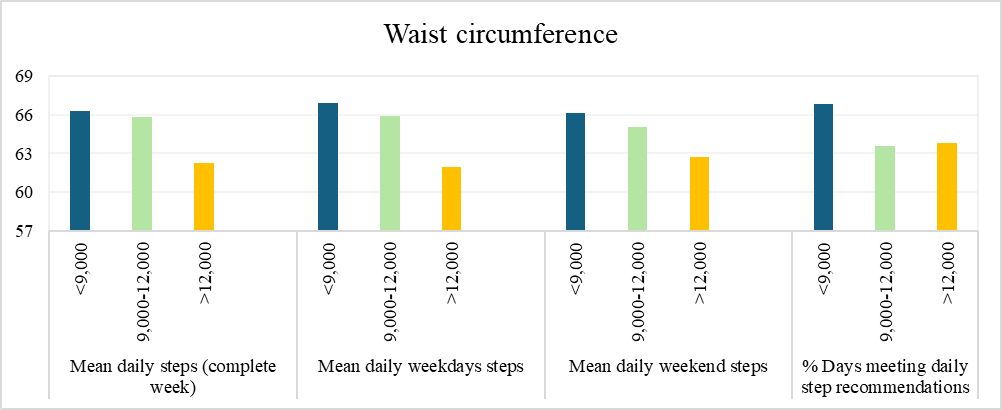
***

# **Figure S4.** Mean differences in body mass index, percentage of body fat, and waist circumference according to categories of mean daily steps in a complete week, on weekdays, and on weekend, and percentage of days meeting daily step recommendations, controlling for age, sex, and number of weeks with step data. The line indicates significant differences in the means (p < 0.05).

***Association with body mass index***


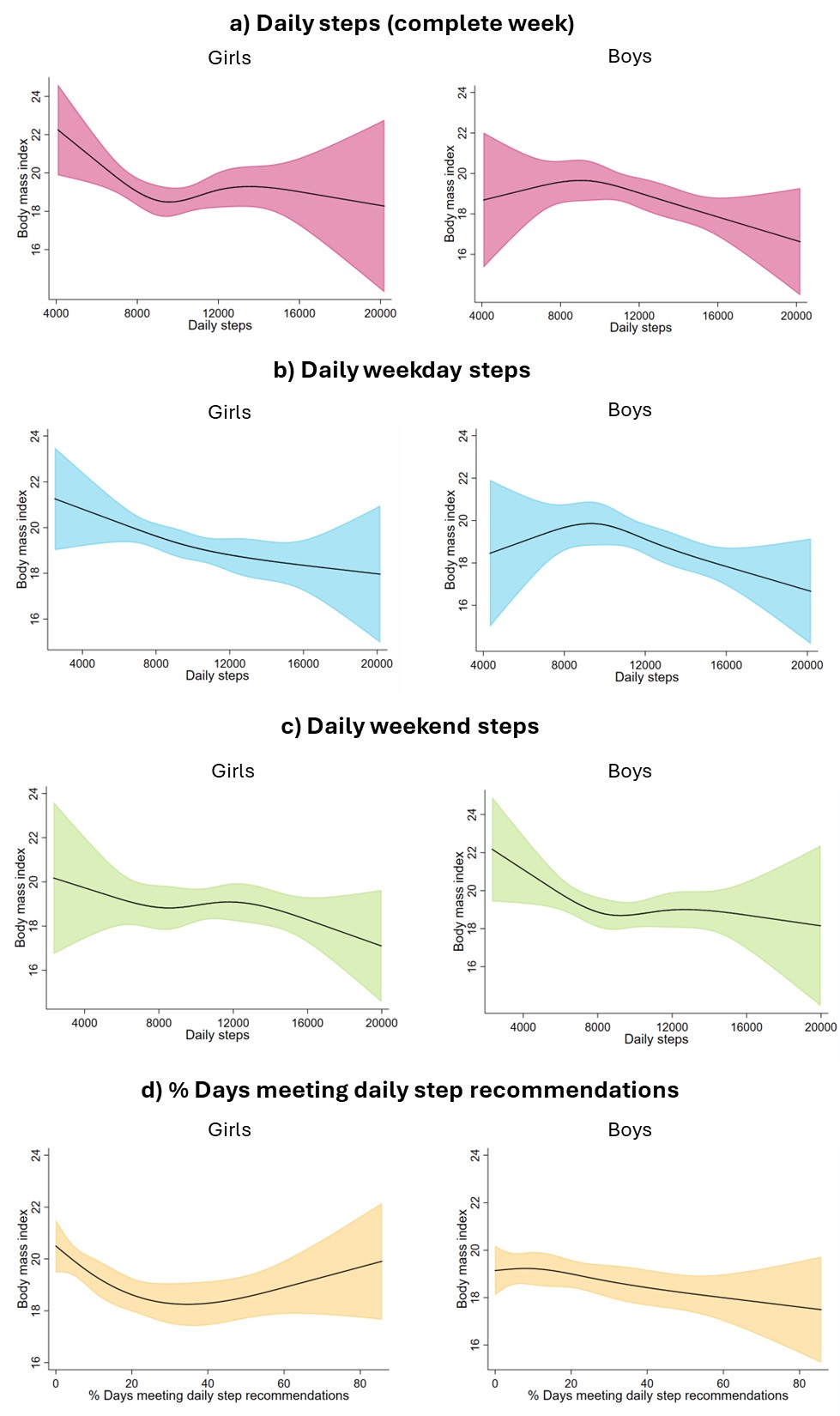


# **Figure S5.** Restricted cubic splines with 95% confidence interval for the association of daily steps and percentage of days meeting daily step recommendations with body mass index (kg/m2) by sex, controlling for age and number of weeks with step data.

***Association with percentage of body fat***


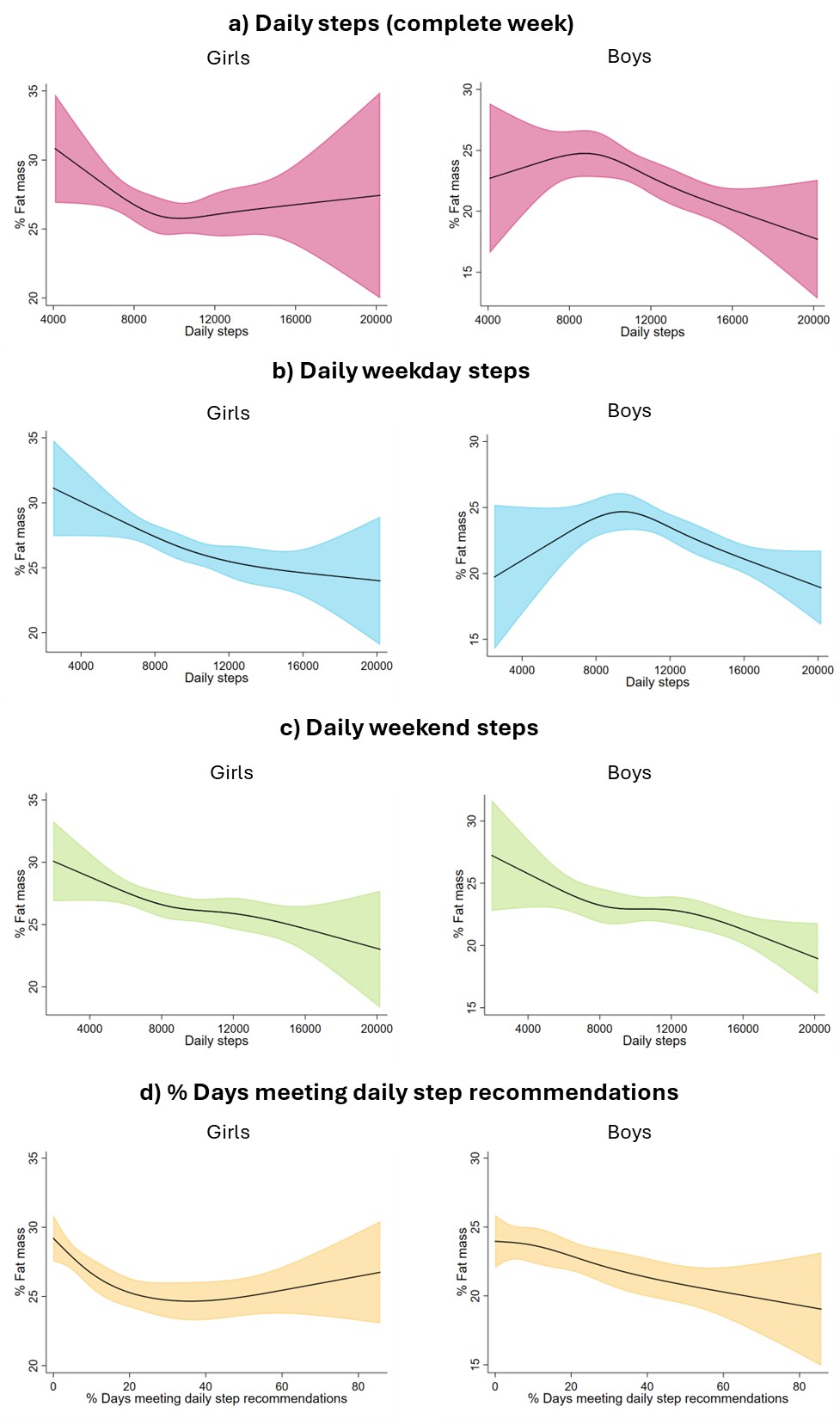


# **Figure S6.** Restricted cubic splines with 95% confidence interval for the association of daily steps and percentage of days meeting daily step recommendations with percentage of body fat by sex, controlling for age and number of weeks with step data.

***Association with waist circumference***


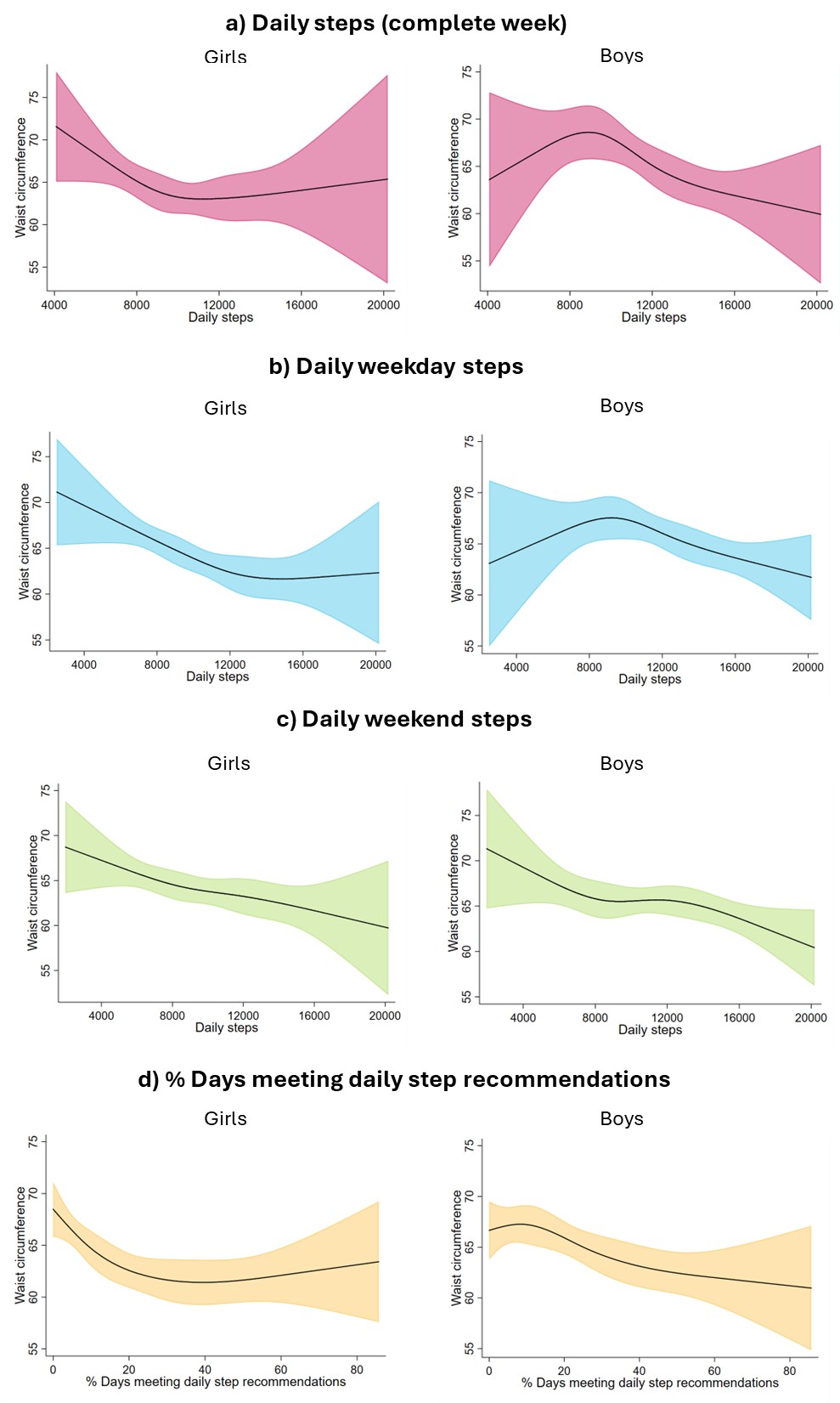


# **Figure S7.** Restricted cubic splines with 95% confidence interval for the association of daily steps and percentage of days meeting daily step recommendations with waist circumference (cm) by sex, controlling for age and number of weeks with step data.


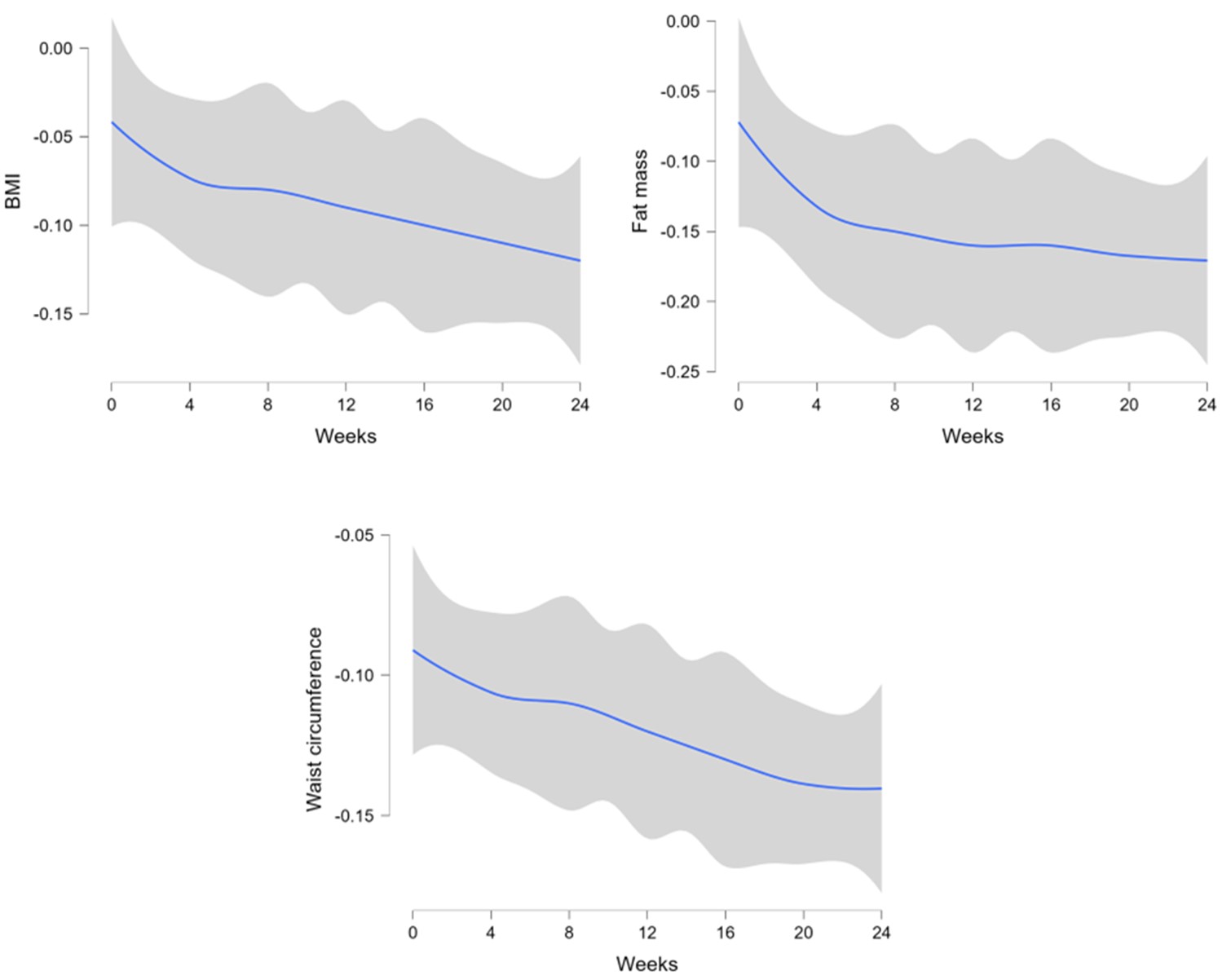


# **Figure S8.** LOESS regression with 95% confidence interval for partial correlation coefficients (r) of daily steps during the complete week with adiposity parameters, controlling for age and sex **by number of weeks of follow-up accumulated (1 to 24 weeks).**


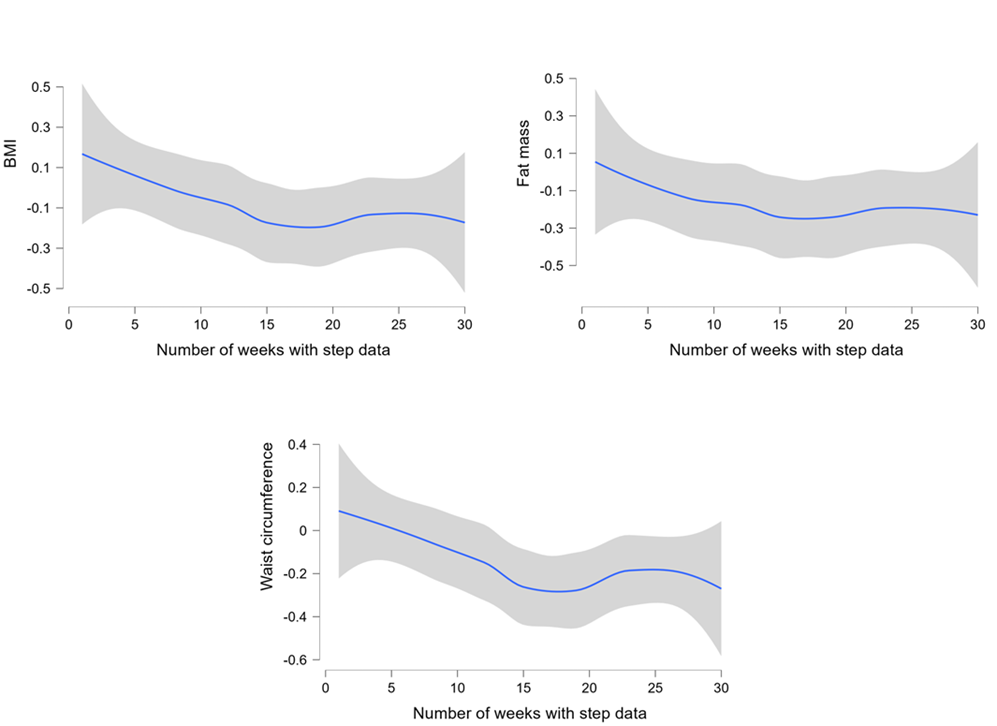


# **Figure S9.** LOESS regression with 95% confidence interval for partial correlation coefficients (r) of daily steps during the complete week with adiposity parameters, controlling for age and sex **by number of weeks with step data.**
